# Distribution and Epidemiological Characteristics of Published Individual Patient Data Meta-Analyses

**DOI:** 10.1371/journal.pone.0100151

**Published:** 2014-06-19

**Authors:** Yafang Huang, Chen Mao, Jinqiu Yuan, Zuyao Yang, Mengyang Di, Wilson Wai-san Tam, Jinling Tang

**Affiliations:** 1 Division of Epidemiology, The Jockey Club School of Public Health and Primary Care, The Chinese University of Hong Kong, Hong Kong SAR, China; 2 The Shenzhen Municipal Key Laboratory for Health Risk Analysis, Shenzhen Research Institute of The Chinese University of Hong Kong, Shenzhen, Guangdong Province, China; National Taiwan University, Taiwan

## Abstract

**Background:**

Individual patient data meta-analyses (IPDMAs) prevail as the gold standard in clinical evaluations. We investigated the distribution and epidemiological characteristics of published IPDMA articles.

**Methodology/Principal Findings:**

IPDMA articles were identified through comprehensive literature searches from PubMed, Embase, and Cochrane library. Two investigators independently conducted article identification, data classification and extraction. Data related to the article characteristics were collected and analyzed descriptively. A total of 829 IPDMA articles indexed until 9 August 2012 were identified. An average of 3.7 IPDMA articles was published per year. Malignant neoplasms (267 [32.2%]) and circulatory diseases (179 [21.6%]) were the most frequently occurring topics. On average, each IPDMA article included a median of 8 studies (Interquartile range, IQR 5 to 15) involving 2,563 patients (IQR 927 to 8,349). Among 829 IPDMA articles, 229 (27.6%) did not perform a systematic search to identify related studies. In total, 207 (25.0%) sought and included individual patient data (IPD) from the “grey literature”. Only 496 (59.8%) successfully obtained IPD from all identified studies.

**Conclusions/Significance:**

The number of IPDMA articles exhibited an increasing trend over the past few years and mainly focused on cancer and circulatory diseases. Our data indicated that literature searches, including grey literature and data availability were inconsistent among different IPDMA articles. Possible biases may arise. Thus, decision makers should not uncritically accept all IPDMAs.

## Introduction

Meta-analysis is a crucial tool in evidence-based medicine because it quantitatively combines results from relevant studies on specific clinical topics, such as treatment effectiveness [1], [2]. Meta-analysis produces results with increased statistical power and minimized bias by integrating data from different studies [3]. Clinicians, treatment guideline developers and medical policy makers often use up-to-date high-quality meta-analyses to support clinical strategies [4].

Meta-analyses are conducted through either aggregate data (AD) or individual patient data (IPD) [5], [6]. AD meta-analyses (ADMAs) are based on group-level results of studies [7], whereas IPD meta-analyses (IPDMAs) collect and integrate individual data from researchers of original primary studies [8]. IPDMA is generally believed to have advantages over ADMA because IPDMA uses consistent inclusion and exclusion criteria among IPD, thus increasing data sensitivity and specificity with detailed data analysis [2], [9], [10]. Therefore, IPDMA is considered as the gold standard in meta-analyses [5], [11].

The number of meta-analyses significantly increased over the past few years, and most meta-analyses were ADMAs [12]. Many studies have documented the characteristics of ADMA articles, such as publication year, study design, and number of studies included [4], [13]. However, studies on prevailing trends and epidemiological characteristics of IPDMA articles are relatively few and are still based on several convenient samples of IPDMAs [14]–[17]. Meta-analysts and clinicians may be unaware of the general trends, prevailing distributions, qualities, and epidemiological characteristics of published IPDMA articles in their relative fields. Moreover, detailed information on the data identification and collection process is required because such information may affect the completeness of the data [16], [18].

This work investigates the distribution and epidemiological characteristics of IPDMA articles indexed until 9 August 2012. This survey on published IPDMA articles may provide important epidemiological information for meta-analytical researchers and clinicians of evidence-based medicine.

## Materials and Methods

### Definition

An article was classified as an IPDMA article if it stated that individual-level data across multiple studies were collected and pooled from original studies.

### Search

We developed a search strategy that combines IPD keywords and five balanced search terms of Montori in searching for IPDMA articles [19]. PubMed, Embase and Cochrane library were searched. The detailed search strategy is given in File S1. In addition, the search strategy developed by Riley et al [5] was used to identify additional eligible IPDMA articles. We also screened the reference lists of all potentially included full-text IPDMAs. No limitation was placed to the year of publication so as to increase the search sensitivity. The latest search was conducted on 9 August 2012.

### Eligibility of IPDMA Articles

No restriction was placed on disease types under investigation or study design. Methodological articles, review protocols and review overviews were excluded. Conference abstracts for which full text articles could not be retrieved were excluded. Non-English articles were also excluded. The most recent articles were included in case of obvious duplication.

### Screening

Two authors independently assessed potentially relevant articles for eligibility. The decision on possible inclusion or exclusion of a study was initially based on the study title, abstract, and then on the full text of articles. Disagreement between the two researchers was resolved by consensus or by consulting a third reviewer if a consensus was not reached.

### Data Extraction and Classification

Data with respect to the epidemiological characteristics of all IPDMA articles were extracted using a form comprising 17 questions, such as publication year, number of included studies and patients, how reviewers identified the studies, and what proportion of request studies actually provided raw data.

Journals that published IPDMA articles were classified by subject category and by impact factors, according to the Thomson Reuters (ISI) Web of Knowledge in 2011 [20]. Impact factors of the journals were divided into three groups, ≥10, ≥5 but <10, and <5. The funding sources were classified into five categories as follows: no funding, non-profit sources (such as government or universities), profit sources (such as pharmaceutical companies), mixed, and unclear. The focus of IPDMA articles was classified into three categories according to the following primary objectives: therapeutic (IPDMA articles studied the effect of treatment or prevention of specific diseases or health conditions) [4], prognosis [17], and others. Diseases cited in IPDMA articles were classified according to the 10th Revision of the International Statistical Classification of Diseases and Related Health Problems (ICD-10) [21]. Studies in IPDMA articles were classified into three categories according to the following methodological design: randomized controlled trials (RCTs), observational studies (cohort or case control studies, or mixed), and others.

Classification and data extraction were independently conducted by two investigators. Discrepancies were resolved through consensus or by consulting a third reviewer if the two investigators failed to research a consensus.

### Statistical Analysis

Descriptive analyses were conducted. Collected data were summarized based on frequencies, median, and interquartile range (IQR). All analyses were conducted using SPSS (version 18.0 for Windows).

## Results

### Search

**Figure 1:**
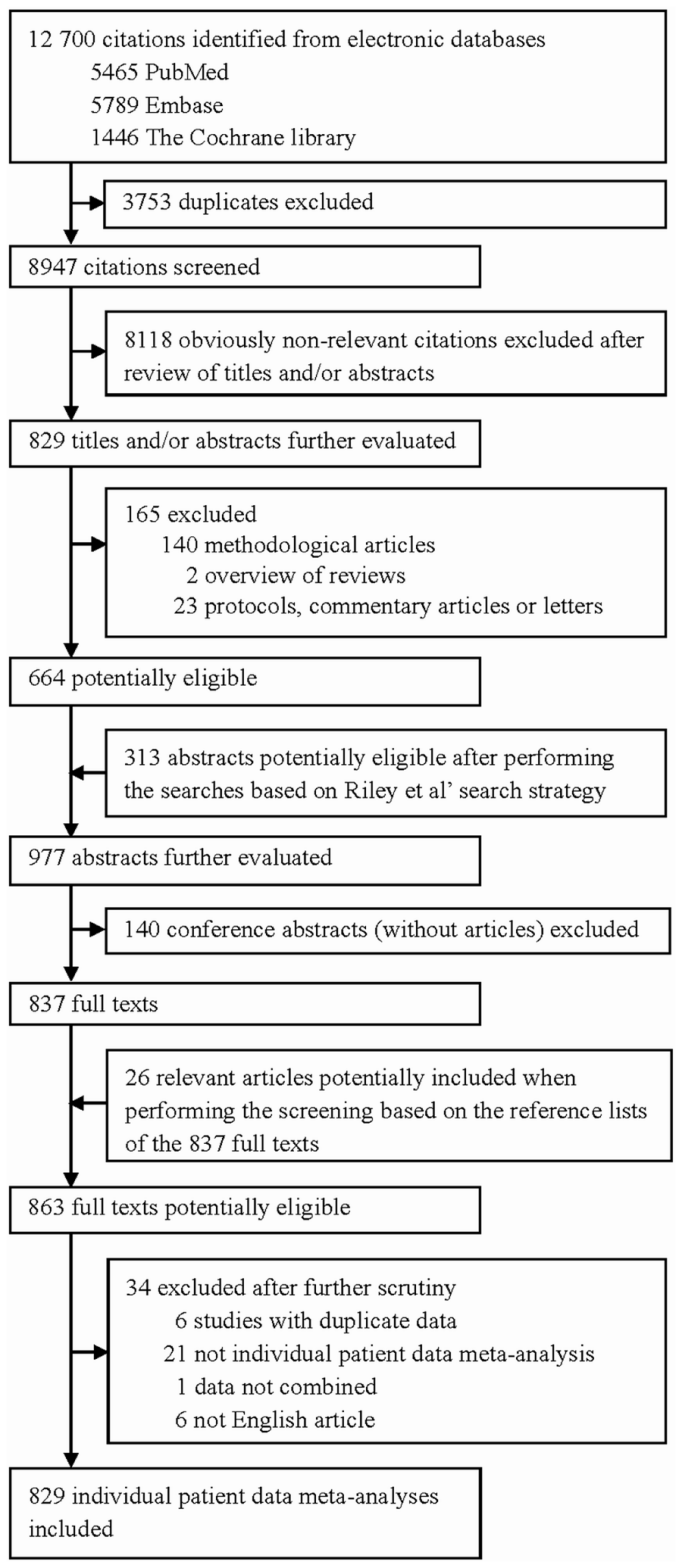
Flowchart of the literature search.

The flowchart of the literature search for IPDMA articles is shown in Figure 1. The initial search identified 12,700 citations from PubMed, Embase and Cochrane Library A total of 664 abstracts were considered potentially eligible after screening the titles and/or abstracts. The search based on the strategy of Riley et al. yielded 313 additional eligible abstracts [5]. A total of 977 abstracts were evaluated further. However, only 837 full texts were retrieved after 140 conference abstracts were excluded. Screening the reference lists of the 837 full texts identified 26 additional potentially eligible studies. Eventually, 34 articles were excluded after further scrutiny. The final count of eligible IPDMA articles included in the study was 829 (the list of the 829 IPDMA articles is given in File S2).

### Epidemiological Description of Published IPDMA Articles

**Figure 2:**
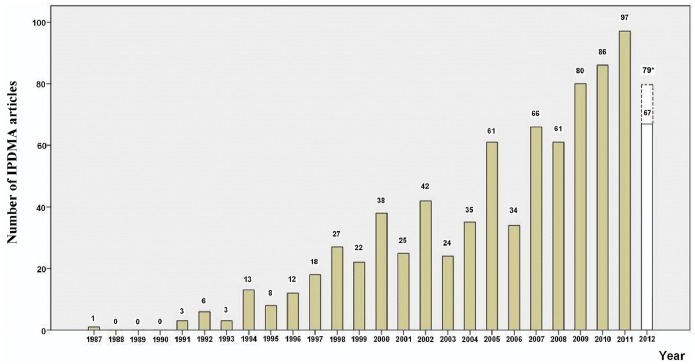
Numbers of IPDMAs published by year up to August 9, 2012. *Expected total numbers of IPDMAs published in 2012.

The distribution of all identified 829 IPDMA articles against the year of publication is presented in Figure 2, which shows an annual average of 31.9 (829/26) IPDMA articles. A regression was fitted for the number of IPDMA articles against year of publication. A slope of 3.7 (*P*<0.001) indicates an average growth of 3.7 IPDMA articles per year (Figure 2).

Table 1 summarizes the 829 IPDMA articles distributed in 287 journals and their epidemiological characteristics. Following the ISI citation report categories, the published journals under “Oncology and hematology” and “Cardiac and cardiovascular system” categories had 256 (30.9%) IPDMA articles, whereas journals under the “Medicine, general and internal” category published 215 (25.9%) IPDMA articles. A total of 264 (31.8%) IPDMA articles were published in journals with high impact factor (≥10). The IPDMA articles had a median of 8 authors (IQR 5–12); approximately half of the corresponding authors (392 [47.2%]) were from the UK and the US. A total of 603 (72.8%) IPDMA articles received funding, 48 (5.8%) did not receive funding and 178 (21.5%) IPDMAs did not report receiving funding.

**Table 1:** Descriptive characteristics of the 829 IPDMAs.

Category	Descriptive Characteristics	N (%)
**General information**		
Journal subject category	Medicine, general and internal	215 (25.9)
	Oncology and hematology	164 (19.8)
	Cardiac and cardiovascular system	92 (11.1)
	Others	358 (43.2)
Journal impact factor	≥10	264 (31.8)
	5–10	220 (26.5)
	<5	307 (37.0)
	Not clear	38 (4.6)
Number of authors included	>15	96 (11.6)
	11–15	117 (14.1)
	6–10	268 (32.3)
	≤5	200 (24.1)
	No individual author listed	148 (17.9)
Country of corresponding author	United Kingdom	225 (27.1)
	United State	167 (20.1)
	France	71 (8.6)
	Netherlands	59 (7.1)
	Canada	53 (6.4)
	Italy	45 (5.4)
	Germany	44 (5.3)
	Australia	41 (4.9)
	Belgium	20 (2.4)
	Japan	18 (2.2)
	Switzerland	16 (1.9)
	Denmark	11 (1.3)
	New Zealand	11 (1.3)
	Others	48 (5.8)
Funding sources	Non-profit supported	415 (50.1)
	Profit sponsor supported	129 (15.6)
	Mixed with profit and non-profit	59 (7.1)
	Declare no funding	48 (5.8)
	Not clear	178 (21.5)
**Clinical information**		
Focus of IPDMA articles	Therapeutic	530 (63.9)
	Prognosis	261 (31.5)
	Others	38 (4.6)
Specialty of diseases according to ICD-10	Malignant neoplasms	267 (32.2)
	Diseases of the circulatory system	179 (21.6)
	Infections and parasitic diseases	59 (7.1)
	Diseases of the nervous system	57 (6.9)
	Mental and behavioral disorders	52 (6.3)
	Diseases of the musculoskeletal system and connective tissue	29 (3.5)
	Endocrine, nutritional and metabolic diseases	21 (2.5)
	Diseases of the genitourinary system	18 (2.2)
	Diseases of the respiratory system	18 (2.2)
	Pregnancy, childbirth and the puerperium	18 (2.2)
	Diseases of the digestive system	16 (1.9)
	Symptoms and signs	16 (1.9)
	Others	79 (9.5)
**Other information**		
Design of studies included in IPDMA article	Randomized controlled trials	505 (60.9)
	Observational studies	114 (13.8)
	Others	210 (25.3)
Type of data	Survival	314 (37.9)
	Binary	298 (35.9)
	Continue	217 (26.2)

Abbreviations: ICD-10, International Statistical Classification of Diseases and Related Health Problems 10th Revision.

Therapeutic IPDMA articles (530 [63.9%]) comprising the majority of the total IPDMA articles outnumbered the prognosis IPDMA articles (261 [31.5%]) and others (38 [4.6%]). The first and second most frequent categories were “malignant neoplasms” [267 (32.2%)] and “diseases of the circulatory system” [179 (21.6%)] according to the category of diseases in ICD-10. The 829 IPDMA articles included over 11,000 independent primary studies with IPD, including approximately 18 million subjects. The median of studies was 8(IQR 5 to 15), and that for patients was 2,563 (IQR 927 to 8,349) for each IPDMA article. A total of 505 (60.9%) IPDMA articles included only RCTs, which outnumbered those that observational studies (114 [13.8%]) and others (210 [25.3%]). In total, 298 (35.9%) IPDMA articles used binary data.

### Factors of the IPDMA Reviewers that may Affect the Completeness of Data

Table 2 summarizes the potential factors associated with the completeness of data in the 829 IPDMA articles. A total of 497 (60.0%) IPDMA articles clearly stated that systematic searches were performed to identify relevant studies, whereas 103 of the remaining 332 (40.0%) IPDMA articles did not state how the studies were searched, and 229 (27.6%) identified the studies based on a selective or non-systematic approach.

**Table 2:** Potential factors associated with the completeness of the data in the 829 IPDMAs.

Category	Characteristics	N (%)
Whether performed a systematic search	Clearly stated based on systematic search approach	497 (60.0)
	Clearly stated based on selective, non-systematic approach	229 (27.6)
	Search approach not clearly stated	103 (12.4)
Whether included “grey literature”*	Sought and included “grey literature”	207 (25.0)
	Sought but not included “grey literature”	127 (15.3)
	Not sought “grey literature”	376 (45.5)
	Not clear	119 (14.3)
IPD collected from all requested studies	Yes	496 (59.8)
	No	296 (35.7)
	Not clear	37 (4.5)
Percentage of studies obtained IPD	100%	496 (59.8)
	≥80%, <100%	86 (10.4)
	≥60%, <80%	105 (12.7)
	≥40%, <60%	61 (7.4)
	≥20%, <40%	27 (3.3)
	<20%	13 (1.6)
	Percentage cannot be calculated	41 (4.9)
Number of studies provided IPD	>20	142 (17.1)
	16–20	55 (6.6)
	11–15	131 (15.8)
	6–10	221 (26.7)
	1–5	271 (32.1)
	Not clear	9 (1.1)
Percentage of IPD obtained	100%	496 (59.8)
	≥80%, <100%	29 (3.5)
	≥60%, <80%	23 (2.8)
	≥40%, <60%	15 (1.8)
	≥20%, <40%	15 (1.8)
	<20%	6 (0.7)
	Percentage cannot be calculated	245 (29.6)
Number of IPD provided	>5000	265 (32.0)
	3001–5000	98 (11.8)
	1001–3000	235 (28.3)
	≤1000	209 (25.2)
	Not clear	22 (2.7)

*“grey literature” indicate unpublished studies, studies reported as meeting abstracts, book chapters, letters or studies published in non-English language journals.

A total of 334 (40.3%) IPDMA articles reported seeking IPD in the “grey literature”, and 207 (25.0%) integrated the results of “grey literature” into their meta-analyses. In total, 376 (45.5%) IPDMA articles reported that they did not seek “grey literature”, whereas 119 (14.3%) IPDMA articles did not report sought in the “grey literature”.

A total of 792 (95.5%) IPDMA articles requested IPD from all identified studies. Nevertheless, only 496 (59.8%) obtained IPD from all requested studies. Thirty-seven (4.5%) of the IPDMA articles did not clearly report whether they requested all studies for IPD.

Among the abovementioned 497 (60.0%) IPDMA articles for which a systematic search was performed, only 190 (38.2%) obtained IPD from all requested studies, whereas 277 (55.7%) did not obtain IPD from all eligible studies. Thirty (6.1%) IPDMA articles did not state whether IPD was obtained from all requested studies.

A total of 788 (95.1%) IPDMA articles provided information on the sum of studies with and without IPD (additionally extracted the information from relative AD) in each article, which enables the determination of the proportion of studies providing IPD among the total studies within each article. Of the 788 IPDMA articles, 582 obtained IPD, comprising up to 80% or more of the total studies. The percentage of studies providing IPD within an IPDMA article had a 100% median (IQR 77.8% to 100%). Meanwhile, the investigators sent mails to the authors of 190 articles requesting for IPD, but only 6 (3.2%) agreed to provide their raw data [22].

A total of 584 (70.4%) IPDMA articles provided information on the total number of patients with and without IPD. Hence, the total proportion of patients with IPD can be calculated. Of the 584 IPDMA articles, 525 obtained 80% or more of the total original IPD. The percentage of patient data in IPD ranged from 4.6% [23] to 100%, with a median of 100% (IQR 100% to 100%).

## Discussion

Our study shows that an increasing number of IPDMA articles are published yearly. This increasing number is attributed to strong information supports, such as Cochrane and high-impact general medical journals, such as *BMJ* and *Annals of Internal Medicine*, for sharing IPD among researchers in recent years [24]. A recent survey found that only 24% of the meta-analysts who attempted to seek IPD resulted in no IPD [12]. However, the number of IPDMA articles remains far less than that of ADMA articles [12], [14]. Among all published articles on meta-analyses, the proportions of IPDMA articles and ADMA articles are 4% and 96% respectively [12]. Nevertheless, with the increase in investigator demand for shared data and the willingness of trialists to self-encourage data sharing [24], the number of IPDMA articles can continuously increase.

Cancer is historically the most prevalent topic in IPDMA articles. Of 34 IPDMA articles, 19 (55.9%) were on the cancer field before 1996 [8]. IPDMA articles for diseases of the circulatory system have made a rapid progress, although cancer remains the most frequent topic for IPDMA articles. In our study, approximately one-third (267 [32.2%]) of the IPDMA articles are in the cancer field, and more than one-fifth (179 [21.6%]) of the IPDMA articles are in the field of circulatory systematic disease. In total, IPDMA articles in these two diseases comprised more than half of all IPDMA articles.

IPDMA articles are more time-consuming and normally require more human resources than ADMA articles [16]. This study shows that the median number of authors in IPDMA articles is eight, a value that is twice that of ADMA articles [4]. IPDMA articles are more likely to be supported by funding from profit sponsors. In our study, 129 (15.6%) of 829 IPDMA articles are supported by profit sponsors. Previous studies show that only 2.3% of the ADMA articles received sponsor support [4]. Compared with 32.2% of IPDMA articles in the cancer field being supported by profit sponsors, the percent of ADMA articles on the cancer field with sponsor support is only 11% [4].

Our study finds that each IPDMA article includes a median of 8 studies (IQR 5 to 15) and a median of 2,563 patients (IQR 927 to 8,349) on average. Previous studies reported that ADMA articles included a median of 16 studies (IQR 7 to 30) and a median of 1,112 patients (IQR 322 to 3,750) [4]. The number of studies in IPDMA articles is smaller than that in ADMA articles. One potential reason for this difference is that IPDMA articles lack systematic searches to identify all relevant studies. However, the number of patients in IPDMA articles is larger than that in ADMA articles. For IPDMA articles, a larger quantity of sensitive studies is derived from systematic searches that often temporize a higher degree of specificity of individual patient data because of clinically more consistent inclusion and exclusion criteria.

Our study finds that 37.9% of IPDMA articles are based on survival data. A previous study showed that only 4.0% of ADMA articles use survival data [13]. IPDMA has some advantages over ADMA because of its intrinsic attributes. One advantage is that IPDMAs are more flexible than ADMA in conducting analyses both clinically and statistically, particularly in dealing with survival outcomes. Survival data identify whether and when an outcome (e.g., death) has occurred [25]. Survival analysis is critical in evaluating therapeutic effects and prognosis in the cancer field [2].

Results suggest that selection bias may affect the completeness of the data. Our result shows that 332 (40.0%) IPDMA articles do not clearly state whether systematic searches were performed to identify relevant studies (103 did not state how they searched for studies, whereas 229 identified the studies based on a selective, or non-systematic approach). Selection bias was a potential concern for IPDMA articles that did not perform a systematic literature search to identify relevant studies. For example, Davidson et al [26] published an IPDMA article to compare biphasic insulin aspart 30 (BIAsp 30) with biphasic human insulin 30 (BHI 30). In the IPDMA article, they only searched the databases of a pharmaceutical company and included six trials for the meta-analysis of major hypoglycemia. The overall OR estimate was 0.45 (95% CI 0.22 to 0.93), which verifies that the likelihood of major hypoglycemia was significantly lower with BIAsp 30 than with BHI 30. However, in an article of ADMA [27], the authors conducted a systematic search comprising nine trials. The overall RR estimate was 0.66 (95% CI 0.31 to 1.41), which was insignificant.

IPDMA articles should also emphasize publication bias. Our result shows that 334 (40.3%) IPDMA articles reported seeking IPD from studies in the “grey literature” and 207 integrated results from the “grey literature” into their meta-analyses. A total of 376 (45.5%) IPDMA articles reported that they did not seek “grey literature”. Given that IPDMA included a higher number of patients, but smaller number of studies than ADMAs, IPD meta-analysts are more likely to seek large published studies on IPD, rather than unpublished small studies. Small unpublished studies may either be omitted because IPD meta-analysts cannot obtain the original data from small studies or because they select large studies while neglecting to perform systematic searches. The omission of small unpublished studies may result in an exaggeration of the risk estimate [28]. Publication bias will more likely occur when only large published studies are sought. Hence, unpublished studies are strongly suggested to be sought in future IPDMAs. If IPD from small unpublished studies is unavailable, IPD meta-analysts can collect related AD and include AD in their estimation. Future IPD meta-analysts should assess publication bias through funnel plot because such investigations are still rare in IPDMAs [18].

Moreover, the bias of IPDMA articles may be derived from data unavailability. Our study found that 582 (70.2%) IPDMAs obtained IPD for 80% or more of the total studies from which IPD were sought. Previous studies on the availability of IPD found that 79% of 142 IPDMA articles published until 2005 obtained IPD for 80% or more of the total studies [5], and 67% of 31 IPDMA articles published between 2007 and 2009 obtained IPD for 80% or more of the total studies [18]. Data availability bias was a potential concern if the meta-analysts failed to obtain IPD from all requested studies [29]. For example, Choy et al. [30] performed an IPDMA study to compare stapled ileocolic anastomoses with handsewn methods, and the overall anastomotic leak was the primary end point. Seven RCTs were identified, and IPD were sought from these studies, but only three out of seven obtained IPD. The fixed effects meta-analysis of the three RCTs with IPD gave an OR of 0.18 (95% CI 0.03 to 1.03; I^2^ = 0%). This result indicated that the stapled method was not associated with low overall anastomotic leaking. When the additional four RCTs that do not provide IPD are included, the fixed effects meta-analysis of all seven trials (three of IPD and four of AD) showed an OR of 0.48 (95% CI 0.24 to 0.95; I^2^ = 21%), which indicated that stapled ileocolic anastomoses were associated with low overall anastomotic leak. Consequently, studies without IPD potentially affect the conclusions. AD was suggested to be collected and added to the calculation if the IPD meta-analysts cannot obtain IPD from all requested studies.

### Strengths

IPDMAs are considered as the gold standard in supporting clinical decision making. This article is the first to conduct a comprehensive search in a cross section study of the distribution and epidemiological characteristics of the published IPDMA articles.

### Limitations

This study has several limitations. First, our data were based on the information reported in published IPDMA articles. The original review authors were not directly contacted. Some details may possibly be omitted (e.g., some publications may have been granted sponsorship but were not reported). Second, we excluded 140 conference abstracts for which full text articles could not be retrieved and this fail to provide detailed information for data extraction. Given that most of these abstracts will be published as full-text journal articles, the database should be updated in the future. Third, direct comparisons of the characteristics between IPDMAs and ADMAs may be inappropriate because the screening conditions of this study are different from those of previous studies. However, these comparisons are limitedly discussed in this study. Moreover, the conclusions in this study were obtained from the cross-sectional data collected from IPDMAs, rather than by comparing the results of this study with those of a previous study.

## Conclusions

This study provides a survey of published IPDMA articles in terms of prevailing distribution and epidemiological characteristics. The number of IPDMA articles is augmented yearly. IPDMA articles on cancer and circulatory diseases comprise more than half of the total IPDMA articles. Meta-analysts mainly focus on therapeutic IPD and minimally focus on prognosis and others. Systematic searches are not often performed. IPD from grey literature are usually not included. IPD are often unavailable. Selection bias, publication bias, and data availability in IPDMAs should be considered and emphasized. Decision makers should be aware of the potential biases in IPDMAs before accepting their results.

## Supporting Information

File S1 **Search strategies.**
(DOC)

File S2 **List of the 829 IPDMA articles.**
(DOC)

## References

[1] Villar J, Carroli G, Belizán JM (1995) Predictive ability of meta-analyses of randomised controlled trials. Lancet 345: 772–776. 7891492 10.1016/s0140-6736(95)90646-0

[2] Lyman G, Kuderer N (2005) The strengths and limitations of meta-analyses based on aggregate data. BMC Med Res Methodol 5: 14. 15850485 10.1186/1471-2288-5-14 PMC1097735

[3] Harvey I, Peters T, Toth B, Stewart L, Parmar MB, et al (1993) Meta-analysis. Lancet 341: 964–965. 8096300

[4] Moher D, Tetzlaff J, Tricco AC, Sampson M, Altman DG (2007) Epidemiology and Reporting Characteristics of Systematic Reviews. PLoS Med 4: e78. 17388659 10.1371/journal.pmed.0040078 PMC1831728

[5] Riley RD, Simmonds MC, Look MP (2007) Evidence synthesis combining individual patient data and aggregate data: a systematic review identified current practice and possible methods. J Clin Epidemiol 60: 431–439. 17419953 10.1016/j.jclinepi.2006.09.009

[6] Riley RD, Lambert PC, Staessen JA, Wang J, Gueyffier F, et al (2008) Meta-analysis of continuous outcomes combining individual patient data and aggregate data. Stat Med 27: 1870–1893. 18069721 10.1002/sim.3165

[7] Lau J, Ioannidis JPA, Schmid CH (1998) Summing up evidence: one answer is not always enough. Lancet 351: 123–127. 9439507 10.1016/S0140-6736(97)08468-7

[8] Stewart LA, Tierney JF, Clarke M (2008) Reviews of individual patient data. In: Higgins JPT, Green S, editors. Cochrane Handbook for Systematic Review of Interventions. Chichester (UK) : John Wiley & Sons, Ltd. 18.1–18.9.

[9] Cooper H, Patall EA (2009) The relative benefits of meta-analysis conducted with individual participant data versus aggregated data. Psychol Methods 14: 165–176. 19485627 10.1037/a0015565

[10] Tierney JF, Stewart LA (2005) Investigating patient exclusion bias in meta-analysis. Int J Epidemiol 34: 79–87. 15561753 10.1093/ije/dyh300

[11] Stewart LA, Parmar MKB (1993) Meta-analysis of the literature or of individual patient data: is there a difference? Lancet 341: 418–422. 8094183 10.1016/0140-6736(93)93004-k

[12] Kovalchik SA (2012) Survey finds that most meta-analysts do not attempt to collect individual patient data. J Clin Epidemiol 65: 1296–1299. 22981246 10.1016/j.jclinepi.2012.07.010 PMC3478473

[13] Davey J, Turner R, Clarke M, Higgins J (2011) Characteristics of meta-analyses and their component studies in the Cochrane Database of Systematic Reviews: a cross-sectional, descriptive analysis. BMC Med Res Methodol 11: 160. 22114982 10.1186/1471-2288-11-160 PMC3247075

[14] Simmonds MC, Higginsa JPT, Stewartb LA, Tierney J, Clarke M, et al (2005) Meta-analysis of individual patient data from randomized trials: a review of methods used in practice. Clin Trials 2: 209–217. 16279144 10.1191/1740774505cn087oa

[15] Koopman L, van der Heijden GJ, Glasziou PP, Grobbee DE, Rovers MM (2007) A systematic review of analytical methods used to study subgroups in (individual patient data) meta-analyses. J Clin Epidemiol 60: 1002–1009. 17884593 10.1016/j.jclinepi.2007.01.018

[16] Riley RD, Lambert PC, Abo-Zaid G (2010) Meta-analysis of individual participant data: rationale, conduct, and reporting. BMJ 340: c221. 20139215 10.1136/bmj.c221

[17] Abo-Zaid G, Sauerbrei W, Riley RD (2012) Individual participant data meta-analysis of prognostic factor studies: state of the art? BMC Med Res Methodol 12: 56. 22530717 10.1186/1471-2288-12-56 PMC3413577

[18] Ahmed I, Sutton AJ, Riley RD (2012) Assessment of publication bias, selection bias, and unavailable data in meta-analyses using individual participant data: a database survey. BMJ 344: d7762. 22214758 10.1136/bmj.d7762

[19] Montori VM, Wilczynski NL, Morgan D, Haynes RB (2005) Optimal search strategies for retrieving systematic reviews from Medline: analytical survey. BMJ 30: 68. 10.1136/bmj.38336.804167.47 PMC543864 15619601

[20] Thomson Reuters (2011) Journal Citation Reports. ISI Web of Knowledge. Available: http://admin-router.webofknowledge.com/?DestApp=JCR. Accessed 2012 Dec 5.

[21] WHO (2011) International Statistical Classification of Diseases and Related Health Problems 10th Revision. Geneva, Switzerland: World Health Organization. Available: http://www.who.int/classifications/icd/en/. Accessed 2012 Dec 5.

[22] DiGiuro G, Ignjatovic D, Brogger J, Bergamaschi R (2006) How accurate are published recurrences rates after rectal prolapse surgery? A meta-analysis of individual patient data. Am J Surg 191: 773–778. 16720147 10.1016/j.amjsurg.2006.01.030

[23] Kendrick D, Smith S, Sutton AJ, Mulvaney C, Watson M, et al (2009) The effect of education and home safety equipment on childhood thermal injury prevention: meta-analysis and meta-regression. Inj Prev 15: 197–204. 19494100 10.1136/ip.2008.020677

[24] Rathi V, Dzara K, Gross CP, Hrynaszkiewicz I, Joffe S, et al (2012) Sharing of clinical trial data among trialists: a cross sectional survey. BMJ 345: e7570. 23169870 10.1136/bmj.e7570 PMC3502744

[25] Clarke M, Godwin J (1998) Systematic reviews using individual patient data: A map for the minefields? Ann Oncol 9: 827–833. 9789604 10.1023/a:1008468705492

[26] Davidson JA, Liebl A, Christiansen JS, Fulcher G, Ligthelm RJ, et al (2009) Risk for nocturnal hypoglycemiawith biphasic insulin aspart 30 compared with biphasic human insulin 30 in adults with type 2 diabetes mellitus: a meta-analysis. Clin Ther 31: 1641–1651. 19808125 10.1016/j.clinthera.2009.08.011

[27] Davidson J, Vexiau P, Cucinotta D, Vaz J, Kawamori R (2005) Biphasic insulin aspart 30: literature review of adverse events associated with treatment. Clin Ther 27 Suppl B: S75–88. 10.1016/j.clinthera.2005.11.022 16519039

[28] Henmi M, Copas J (2010) Confidence intervals for random effects meta-analysis and robustness to publication bias. Stat Med 29: 2969–2983. 20963748 10.1002/sim.4029

[29] Paule R, Mandel J (1982) Consensus values and weighting factors. J Res Natl Bur Stand 87: 377–385. 10.6028/jres.087.022 PMC6768160 34566088

[30] Choy PYG, Bissett IP, Docherty JG, Parry BR, Merrie A, et al (2007) Stapled versus handsewn methods for ileocolic anastomoses. Cochrane Database Syst Rev 18: CD004320. 10.1002/14651858.CD004320.pub2 17636751

