# Supplementary material for: Distribution and Epidemiological Characteristics of Published Individual Patient Data Meta-Analyses
Source: PLoS One. 2014 Jun 19;9(6):e100151. doi: 10.1371/journal.pone.0100151 (PMC4063791; doi:10.1371/journal.pone.0100151)
Supplement: File S1 — Search strategies. (DOC) [file pone.0100151.s001.doc]

**Supporting Information File S1. Search strategy**

**PubMed search strategy**

| **ID** | **Search** |
| --- | --- |
| #1 | IPD[Title/Abstract] OR Collaborative[Title/Abstract] OR MAP[Title/Abstract] OR individual patient$[Title/Abstract] OR individual participant$[Title/Abstract] OR individual data[Title/Abstract] OR patient$ level[Title/Abstract] OR original data[Title/Abstract] OR original research[Title/Abstract] OR original case[Title/Abstract] |
| #2 | Cochrane Database Syst Rev [TA] OR search [Title/Abstract] OR meta-analysis [Publication Type] OR MEDLINE [Title/Abstract] OR (systematic [Title/Abstract] AND review [Title/Abstract]) |
| #3 | (#1 AND #2) |

**EMBASE search strategy**

| **ID** | **Search** |
| --- | --- |
| #1 | IPD.ti,ab. OR Collaborative.ti,ab. OR MAP.ti,ab. OR individual patient$.ti,ab. OR individual participant$.ti,ab. OR individual data.ti,ab. OR patient$ level.ti,ab. OR original data.ti,ab. OR original research.ti,ab. OR original case.ti,ab. |
| #2 | Cochrane database of systematic reviews.jn. OR search.tw. OR meta-analysis.pt. OR MEDLINE.tw. OR systematic review.tw. |
| #3 | 1 and 2 |

**Cochrane search strategy**

#1 (IPD).ti,ab.

#2 (Collaborative).ti,ab.

#3 (MAP).ti,ab.

#4 (individual patient$).ti,ab.

#5 (individual participant$).ti,ab.

#6 (individual data).ti,ab.

#7 (patient$ level).ti,ab.

#8 (original data).ti,ab.

#9 (original research).ti,ab.

#10 (original case).ti,ab.

#11 (Cochrane database of systematic reviews).jn.

#12 (search).tw.

#13 (meta-analysis).pt.

#14 (MEDLINE).tw.

#15 (systematic review).tw.

#16 (#1 OR #2 OR #3 OR #4 OR #5 OR #6 OR #7 OR #8 OR #9 OR #10)

#17 (#11 OR #12 OR #13 OR #14 OR #15)

#18 (#16 AND #17)

**References**

1. Montori VM, Wilczynski NL, Morgan D, Haynes RB (2005) Optimal search strategies for retrieving systematic reviews from Medline: analytical survey. BMJ 330:68.
