# Supplementary material for: Distribution and Epidemiological Characteristics of Published Individual Patient Data Meta-Analyses
Source: PLoS One. 2014 Jun 19;9(6):e100151. doi: 10.1371/journal.pone.0100151 (PMC4063791; doi:10.1371/journal.pone.0100151)
Supplement: File S2 — List of the 829 IPDMA articles. (DOC) [file pone.0100151.s002.doc]

**Supporting Information File S2. List of the 829 IPDMA articles**

| **Number** | **Journal Name** | **Year, vol** | **Title** |
| --- | --- | --- | --- |
| 1 | Clinical Orthopaedics and Related Research | 2012; 470:567–577 | Nonoperative versus Operative Treatment for Thoracolumbar Burst fractures without neurologic deficit |
| 2 | American Journal of Public Health | 2012; 102(2): 309-318. | Individual Participant Data Meta-Analysis of Mechanical Workplace Risk Factors and Low Back Pain |
| 3 | The Lancet | 2012; 379(9813): 322–334. | Self-monitoring of oral anticoagulation systematic review and meta-analysis of individual patient data. |
| 4 | American Journal of Obstetrics & Gynecology | 2012; 206(2): e121-124. | Vaginal progesterone in women with an asymptomatic sonographic short cervix in the midtrimester decreases preterm delivery and neonatal morbidity: a systematic review and metaanalysis of individual patient data |
| 5 | Lung Cancer | 2012; 76(3):478-485. | Benefits and risks of using erythropoiesis-stimulating agents (ESAs) in lung cancer patients: Study-level and patient-level meta-analyses |
| 6 | The American Journal of Surgery | 2011;201: 695–699. | Malignant eccrine spiradenoma: a meta-analysis of reported cases |
| 7 | Pediatrics | 2011; 128(4): 729-739. | Inhaled nitric oxide in preterm infants: an individual-patient data meta-analysis of randomized trials. |
| 8 | Pharmacoepidemiol Drug Safety | 2011;20(2):119-30. | Cancer risk with tumor necrosis factor alpha (TNF) inhibitors: meta-analysis of randomized controlled trials of adalimumab, etanercept, and infliximab using patient level data |
| 9 | The Journal of Trauma and Acute Care Surgery | 2011;71: 1003–1010. | Recovery after injury: an individual patient data meta-analysis of general health status using the EQ-5D |
| 10 | Radiotherapy and Oncology | 2011;100(1):33-40. | Meta-analysis of chemotherapy in head and neck cancer (MACH-NC): a comprehensive analysis by tumour site. |
| 11 | Journal of Clinical Oncology | 2011; 29:2781-2786 | impact of young age on treatment efficacy and safety in advanced colorectal cancer: a pooled analysis of patients from nine first-line phase III chemotherapy trials |
| 12 | European Journal of Vascular and Endovascular Surgery | 2011;41(2): 153-158. | Age modifies the relative risk of stenting versus endarterectomy for symptomatic carotid stenosis--a pooled analysis of EVA-3S, SPACE and ICSS. |
| 13 | Journal of Clinical Oncology | 2011;29(18): 2590-2597. | Effect of amifostine on survival among patients treated with radiotherapy: a meta-analysis of individual patient data. |
| 14 | Journal of the American College of Cardiology | 2011;58(19): 1945-1954. | Impact of platelet reactivity on clinical outcomes after percutaneous coronary intervention. A collaborative meta-analysis of individual participant data. |
| 15 | Human Reproduction Update | 2011;17(3): 301–310. | Chlamydia antibody testing and diagnosing tubal pathology in subfertile women: an individual patient data meta-analysis |
| 16 | Human Reproduction Update | 2011;17(3): 293-300. | Are patient characteristics associated with the accuracy of hysterosalpingography in diagnosing tubal pathology? An individual patient data meta-analysis. |
| 17 | Blood | 2011; 117(26): 7007-7013. | Individual patient data meta-analysis of randomized trials evaluating IL-2 monotherapy as remission maintenance therapy in acute myeloid leukemia |
| 18 | Annals of Oncology | 2011;22(12): 2604-2609. | Efficacy of capecitabine versus 5-fluorouracil in colorectal and gastric cancers: a meta-analysis of individual data from 6171 patients. |
| 19 | The Lancet Oncology | 2011;12(11): 1023-1031. | Intrauterine device use, cervical infection with human papillomavirus, and risk of cervical cancer: a pooled analysis of 26 epidemiological studies |
| 20 | Journal of Periodontology | 2012;83(4): 477-490. | Evidence-Based Periodontal Plastic Surgery. II. An Individual Data Meta-Analysis for Evaluating Factors in Achieving Complete Root Coverage. |
| 21 | Journal of Clinical Oncology | 2011;29(31): 4199-4204. | Alternative end points to evaluate a therapeutic strategy in advanced colorectal cancer: evaluation of progression-free survival, duration of disease control, and time to failure of strategy--an Aide et Recherche en Cancerologie Digestive Group Study |
| 22 | Cochrane Database of Systematic Reviews | 2011; 7(9): CD004320. | Stapled versus handsewn methods for ileocolic anastomoses. |
| 23 | The Journal of Rheumatology | 2000;27(10): 2424-2431. | A pooled data analysis of the use of intermittent cyclical etidronate therapy for the prevention and treatment of corticosteroid inducted bone loss |
| 24 | Journal of the American College of Cardiology | 2011;57(19):1877-1886. | Central obesity and survival in subjects with coronary artery disease: a systematic review of the literature and collaborative analysis with individual subject data |
| 25 | The Lancet | 2011;378(9804): 1707-1716. | Effect of radiotherapy after breast-conserving surgery on 10-year recurrence and 15-year breast cancer death: meta-analysis of individual patient data for 10,801 women in 17 randomised trials. |
| 26 | The Lancet | 2011;378(9793): 771-784. | Relevance of breast cancer hormone receptors and other factors to the efficacy of adjuvant tamoxifen: patient-level meta-analysis of randomised trials |
| 27 | American Heart Journal | 2011;161(3): 500-507. | High-risk patients with ST-elevation myocardial infarction derive greatest absolute benefit from primary percutaneous coronary intervention: Results from the Primary Coronary Angioplasty Trialist versus Thrombolysis (PCAT)-2 Collaboration. |
| 28 | Journal of Thrombosis & Haemostasis | 2011;9(12): 2361-2370. | Early glycoprotein IIb-IIIa inhibitors in primary angioplasty-abciximab long-term results (EGYPT-ALT) cooperation: individual patient’s data meta-analysis (pages 2361–2370) |
| 29 | Clinical Research in Cardiology | 2011;100(7): 561-570. | Long-term outcome after sirolimus-eluting stents versus bare metal stents in patients with diabetes mellitus: a patient-level meta-analysis of randomized trials |
| 30 | The Lancet Oncology | 2011;12(12): 1134-1142. | HER2 and TOP2A as predictive markers for anthracycline-containing chemotherapy regimens as adjuvant treatment of breast cancer: a meta-analysis of individual patient data. |
| 31 | British Medical Journal | 2011;342: d813. | Risk of recurrence after venous thromboembolism in men and women: patient level meta-analysis. |
| 32 | PLoS One | 2011;6(4): e18801. | Dendritic cell based tumor vaccination in prostate and renal cell cancer: a systematic review and meta-analysis |
| 33 | Blood | 2011;118(5): 1239-1247. | Thalidomide for previously untreated elderly patients with multiple myeloma: meta-analysis of 1685 individual patient data from 6 randomized clinical trials. |
| 34 | Cancer | 2011;117(6): 1262-1271. | Tumor response and progression-free survival as potential surrogate endpoints for overall survival in extensive stage small-cell lung cancer: findings on the basis of North Central Cancer Treatment Group trials. |
| 35 | Schizophrenia research | 2011;126(1-3): 212-219. | Relative indices of treatment effect may be constant across different definitions of response in schizophrenia trials. |
| 36 | PLoS Medicine | 2011;8(1): e1000391 | Development of a standardized screening rule for tuberculosis in people living with HIV in resource-constrained settings: individual participant data meta-analysis of observational studies. |
| 37 | Human Reproduction Update | 2011;17(4): 501-509. | Is aspirin effective in women undergoing in vitro fertilization (IVF)? Results from an individual patient data meta-analysis (IPD MA). |
| 38 | World Neurosurgery | 2011;75(1): 64-72. | True aneurysms of the posterior communicating artery: a systematic review and meta-analysis of individual patient data. |
| 39 | International Forum of Allergy & Rhinology | 2011;1(4): 255-261. | Outcome results of endoscopic vs craniofacial resection of sinonasal malignancies: a systematic review and pooled-data analysis |
| 40 | Journal of Clinical Oncology | 2011;29(13): 1757-1764. | Venous thromboembolic events with chemotherapy plus bevacizumab: a pooled analysis of patients in randomized phase II and III studies. |
| 41 | Circulation | 2011;124(19): 2056-2064. | Isolated low levels of high-density lipoprotein cholesterol are associated with an increased risk of coronary heart disease: an individual participant data meta-analysis of 23 studies in the Asia-Pacific region. |
| 42 | Clinical Cancer Research | 2011;17(9): 2967-2976. | Persistence of disseminated tumor cells in the bone marrow of breast cancer patients predicts increased risk for relapse--a European pooled analysis |
| 43 | Addiction | 2001;96:683-690. | A meta-analysis comparing buprenorphine to methadone for treatment of opiate dependence. |
| 44 | Heart | 2011;97(12): 959-963 | Quantifying the added value of BNP in suspected heart failure in general practice: an individual patient data meta-analysis. |
| 45 | The Lancet Oncology | 2011;12(13): 1229-1239. | Meningiomas in children and adolescents: a meta-analysis of individual patient data |
| 46 | American Heart Journal | 2011;162(4): 740-747. | A meta-analysis of specifically designed randomized trials of sirolimus-eluting versus paclitaxel-eluting stents in diabetic patients with coronary artery disease |
| 47 | Drugs in R&D | 2011;11(3): 277-288 | Short-term acetylsalicylic acid (aspirin) use for pain, fever, or colds - gastrointestinal adverse effects: a meta-analysis of randomized clinical trials |
| 48 | Journal of Thrombosis & Haemostasis | 2011;9(3): 464-472. | Individual patient data meta-analysis of enoxaparin vs. unfractionated heparin for venous thromboembolism prevention in medical patients. |
| 49 | Digestive and Liver Disease | 2011;43(9): 707-713 | Racecadotril for childhood gastroenteritis: an individual patient data meta-analysis |
| 50 | Alcohol and Alcoholism | 2011;46(1): 61-67 | Alcohol-use disorders and depression: results from individual patient data meta-analysis of the acamprosate-controlled studies. |
| 51 | Journal of Pediatrics | 2011;159(4): 644-651 e644. | Procalcitonin is a predictor for high-grade vesicoureteral reflux in children: meta-analysis of individual patient data |
| 52 | The Journal of the American Medical Association | 2011;305(13): 1327-1335 | Low-frequency HIV-1 drug resistance mutations and risk of NNRTI-based antiretroviral treatment failure: a systematic review and pooled analysis |
| 53 | PLoS Medicine | 2011;8(2): e1000416. | Intravaginal practices, bacterial vaginosis, and HIV infection in women: individual participant data meta-analysis. |
| 54 | Cochrane Database of Systematic Reviews | 2011;12: CD004026 | Day hospital versus admission for acute psychiatric disorders |
| 55 | Gut | 2011;60(2): 255-260. | Corticosteroids improve short-term survival in patients with severe alcoholic hepatitis: meta-analysis of individual patient data |
| 56 | Pain | 2011;152(5): 982-989. | Minimum efficacy criteria for comparisons between treatments using individual patient meta-analysis of acute pain trials: examples of etoricoxib, paracetamol, ibuprofen, and ibuprofen/paracetamol combinations after third molar extraction |
| 57 | International Journal of Cancer | 2011;129(3): 713-723 | Biologic markers of sun exposure and melanoma risk in women: pooled case-control analysis. |
| 58 | Journal of Human Hypertension | 2011;29(6): 1034-1042 | Aortic stiffness is reduced beyond blood pressure lowering by short-term and long-term antihypertensive treatment: a meta-analysis of individual data in 294 patients |
| 59 | Circulation | 2011;123(15): 1622-1632. | Clinical Benefit of Statin Pretreatment in Patients Undergoing Percutaneous Coronary Intervention: A Collaborative Patient-Level Meta-Analysis of 13 Randomized Studies. |
| 60 | Cephalalgia | 2002;22(8):633-58 | Triptans (serotonin, 5-HT1B/1D agonists) in migraine: detailed results and methods of a meta-analysis of 53 trials |
| 61 | Osteoporosis International | 2011;22(4): 1199-1209. | Associations of APOE gene polymorphisms with bone mineral density and fracture risk: a meta-analysis. |
| 62 | Cancer | 2011;117(3): 581-589. | Primary cardiac lymphoma: An analysis of presentation, treatment, and outcome patterns |
| 63 | British Medical Journal | 2011;343: d3805 | Glycaemic control in type 1 diabetes during real time continuous glucose monitoring compared with self monitoring of blood glucose: meta-analysis of randomised controlled trials using individual patient data. |
| 64 | Cochrane Database of Systematic Reviews | 2011;(4): CD001081. | Carotid endarterectomy for symptomatic carotid stenosis |
| 65 | Journal of the American College of Cardiology | 2011;58(5): 522-529. | The predictive ability of pre-operative B-type natriuretic peptide in vascular patients for major adverse cardiac events: an individual patient data meta-analysis. |
| 66 | The Lancet | 2011;377(9759): 31-41 | Effect of daily aspirin on long-term risk of death due to cancer: analysis of individual patient data from randomised trials |
| 67 | Journal of Thrombosis & Haemostasis | 2011; 9(2): 312-319 | Risk factors for catheter-related thrombosis (CRT) in cancer patients: a patient-level data (IPD) meta-analysis of clinical trials and prospective studies |
| 68 | Journal of Clinical Endocrinology & Metabolism | 2011;96(5): 1327-1335 | Place of cabergoline in acromegaly: a meta-analysis. |
| 69 | European Journal of Cancer | 2011;47(7): 990-996. | Two or three year disease-free survival (DFS) as a primary end-point in stage III adjuvant colon cancer trials with fluoropyrimidines with or without oxaliplatin or irinotecan: data from 12,676 patients from MOSAIC, X-ACT, PETACC-3, C-06, C-07 and C89803 |
| 70 | Journal of the National Cancer Institute | 1999;91:861-68 | Chemotherapeutic options in chronic lymphocytic leukemia: a meta-analysis of the randomized trials |
| 71 | British Medical Journal | 2011;343: d3952. | Effectiveness of vertebroplasty using individual patient data from two randomised placebo controlled trials: meta-analysis |
| 72 | Nephrology Dialysis Transplantation | 2011;26(3): 848-857. | Early change in proteinuria as a surrogate outcome in kidney disease progression: a systematic review of previous analyses and creation of a patient-level pooled dataset |
| 73 | Auris Nasus Larynx | 2011;8(24): 397-401. | Basaloid squamous cell carcinoma of the larynx-A systematic review. |
| 74 | Cochrane Database of Systematic Reviews | 2011;(7): CD007398. | Intra-aortic balloon pump counterpulsation (IABP) for myocardial infarction complicated by cardiogenic shock. |
| 75 | The Lancet Oncology | 2011;12(6): 540-550. | Sentinel-lymph-node procedure in colon and rectal cancer: a systematic review and meta-analysis |
| 76 | Journal of the American Association for Pediatric Ophthalmology and Strabismus | 2011;15(5): 441-446. | Pediatric optic neuritis and risk of multiple sclerosis: meta-analysis of observational studies |
| 77 | Circulation | 2011;4(1): 18-26. | Efficacy and safety of carvedilol in treatment of heart failure with chronic kidney disease: a meta-analysis of randomized trials |
| 78 | The Lancet | 2010; 375(9709): 132-140. | C-reactive protein concentration and risk of coronary heart disease, stroke, and mortality: an individual participant meta-analysis. |
| 79 | The Lancet | 2011;377(9771): 1085-1095. | Separate and combined associations of body-mass index and abdominal adiposity with cardiovascular disease: collaborative analysis of 58 prospective studies |
| 80 | Journal of the National Cancer Institute | 2011;103(2): 129-142. | Contemporary diagnostic imaging modalities for the staging and surveillance of melanoma patients: a meta-analysis |
| 81 | Journal of the National Cancer Institute | 2011;103(20): 1498-1506. | Outcomes among black patients with stage II and III colon cancer receiving chemotherapy: an analysis of ACCENT adjuvant trials |
| 82 | Stroke | 2011;42(9): 2515-2520. | Safety and functional outcome of thrombolysis in dissection-related ischemic stroke: a meta-analysis of individual patient data |
| 83 | Cochrane Database of Systematic Reviews | 2010;(5): CD007309. | Chemotherapy and supportive care versus supportive care alone for advanced non-small cell lung cancer. |
| 84 | The Lancet | 2010;375(9722): 1267-1277 | Adjuvant chemotherapy, with or without postoperative radiotherapy, in operable non-small-cell lung cancer: two meta-analyses of individual patient data |
| 85 | International Journal of Cardiology | 2010;141(2): 132-140 | Multi-detector computerized tomography angiography for evaluation of acute chest pain--a meta analysis and systematic review of literature |
| 86 | Journal of Clinical Oncology | 2010;28(13): 2181-2190. | Meta-analysis of concomitant versus sequential radiochemotherapy in locally advanced non-small-cell lung cancer |
| 87 | Journal of Thrombosis & Haemostasis | 2010;8(11): 2436-2442 | Does the clinical presentation and extent of venous thrombosis predict likelihood and type of recurrence? A patient-level meta-analysis. |
| 88 | The Lancet | 2010;376(9753): 1670-1681. | Efficacy and safety of more intensive lowering of LDL cholesterol: a meta-analysis of data from 170,000 participants in 26 randomised trials. |
| 89 | Cochrane Database of Systematic Reviews | 2010;(12): CD002026 | Hyperfractionated or accelerated radiotherapy for head and neck cancer |
| 90 | Hypertension | 2010; 56(3): 533-539 | Monitoring initial response to Angiotensin-converting enzyme inhibitor-based regimens: an individual patient data meta-analysis from randomized, placebo-controlled trials |
| 91 | Journal of Pediatric Gastroenterology & Nutrition | 2010;50(1): 79-84 | Infant Formula Supplementation With Long-chain Polyunsaturated Fatty Acids Has No Effect on Bayley Developmental Scores at 18 Months of Age-IPD Meta-analysis of 4 Large Clinical Trials |
| 92 | PLoS Medicine | 2010;7(5): e1000279 | Subtyping of breast cancer by immunohistochemistry to investigate a relationship between subtype and short and long term survival: a collaborative analysis of data for 10,159 cases from 12 studies |
| 93 | British Medical Journal | 2010;341: c3691 | Effect of calcium supplements on risk of myocardial infarction and cardiovascular events: meta-analysis. |
| 94 | The Lancet | 2010;376(9746): 1062-1073. | Short-term outcome after stenting versus endarterectomy for symptomatic carotid stenosis: a preplanned meta-analysis of individual patient data. |
| 95 | The Journal of the American Medical Association | 2010;303(9): 865-873. | Higher vs Lower Positive End-Expiratory Pressure in Patients With Acute Lung Injury and Acute Respiratory Distress Syndrome: Systematic Review and Meta-analysis. |
| 96 | Diabetes | 2010;59(2): 486-494 | Maternal Age at Birth and Childhood Type 1 Diabetes: A Pooled Analysis of 30 Observational Studies |
| 97 | Diabetologia | 2010;53(4): 641-651 | Birthweight and the risk of childhood-onset type 1 diabetes: a meta-analysis of observational studies using individual patient data |
| 98 | Pulmonary Pharmacology and Therapeutics | 2010;23(2): 135-144. | The therapeutic efficacy of erdosteine in the treatment of chronic obstructive bronchitis: a meta-analysis of individual patient data. |
| 99 | British Journal of Haematology | 2010;149(5): 722-733 | Systematic review of the addition of vincristine plus steroid pulses in maintenance treatment for childhood acute lymphoblastic leukaemia - an individual patient data meta-analysis involving 5659 children. |
| 100 | Archives of Internal Medicine | 2010;170(18): 1622-1631. | Effects of Lowering Homocysteine Levels With B Vitamins on Cardiovascular Disease, Cancer, and Cause-Specific Mortality: Meta-analysis of 8 Randomized Trials Involving 37 485 Individuals. |
| 101 | Stroke | 2010;41(11): 2632-2636 | Early mobilization after stroke: an example of an individual patient data meta-analysis of a complex intervention |
| 102 | Breast Cancer Research & Treatment | 2010;119(3): 621-631 | Fifteen-year trends in metastatic breast cancer survival in Greece |
| 103 | Human Reproduction Update | 2010;16(6): 568-576 | Individual patient data meta-analysis of randomized evidence to assess the effectiveness of laparoscopic uterosacral nerve ablation in chronic pelvic pain |
| 104 | JACC cardiovascluar interventions | 2010;3(3): 324-331 | Mortality and morbidity reduction by primary percutaneous coronary intervention is independent of the patient's age |
| 105 | Journal of Clinical Psychiatry | 2001;62(10):757-71 | The effects of olanzapine on the 5 dimensions of schizophrenia derived by factor analysis: combined results of the North American and international trials |
| 106 | Stroke | 2010; 41(6): 1294-1297 | Prevalence of asymptomatic carotid artery stenosis in the general population: an individual participant data meta-analysis. |
| 107 | Otolaryngology - Head & Neck Surgery | 2010;142(2): 155-159. | Postmaneuver restrictions in benign paroxysmal positional vertigo: An individual patient data meta-analysis |
| 108 | European Journal of Cancer | 2010;46(4): 735-743. | Clinical assessment of patients with advanced non-small-cell lung cancer eligible for second-line chemotherapy: a prognostic score from individual data of nine randomised trials. |
| 109 | Annals of Internal Medicine | 2010;153(8): 523-531. | Patient-level meta-analysis: effect of measurement timing, threshold, and patient age on ability of D-dimer testing to assess recurrence risk after unprovoked venous thromboembolism. |
| 110 | Journal of Clinical Oncology | 2010;28(3): 509-518 | Meta-analysis of breast cancer outcomes in adjuvant trials of aromatase inhibitors versus tamoxifen |
| 111 | Cochrane Database of Systematic Reviews | 2010;(5): CD005066. | Stroke liaison workers for stroke patients and carers: an individual patient data meta-analysis |
| 112 | The Journal of the American Medical Association | 2010;303(1): 47-53. | Antidepressant Drug Effects and Depression Severity: A Patient-Level Meta-analysis |
| 113 | Journal of the American College of Cardiology | 2010;55(22): 2435-2445. | Long-term outcome of a routine versus selective invasive strategy in patients with non-ST-segment elevation acute coronary syndrome a meta-analysis of individual patient data. |
| 114 | Cardiol Journal | 2010;17(3): 259-266 | Short-term antihypertensive efficacy of perindopril according to clinical profile of 3,188 patients: A meta-analysis |
| 115 | Arzneimittelforschung | 2010; 60(7): 415-420. | Use of isoxsuprine hydrochloride as a tocolytic agent in the treatment of preterm labour: a systematic review of previous literature. |
| 116 | Kidney Int | 2011;79(12):1341-52 | Lower estimated glomerular filtration rate and higher albuminuria are associated with all-cause and cardiovascular mortality. A collaborative meta-analysis of high-risk population cohorts |
| 117 | Sleep Medicine Reviews | 2010;14(5): 287-297 | Maxillomandibular advancement for the treatment of obstructive sleep apnea: a systematic review and meta-analysis |
| 118 | Diabetes, Obesity and Metabolism | 2010;12(9): 772-779. | Meta-analysis of individual patient data to assess the risk of hypoglycaemia in people with type 2 diabetes using NPH insulin or insulin glargine. |
| 119 | Journal of Thoracic & Cardiovascular Surgery | 2010; 139(6): 1441-1446 | Impact of positive pleural lavage cytology on survival in patients having lung resection for non-small-cell lung cancer: An international individual patient data meta-analysis |
| 120 | Stroke | 2010; 41(4): 624-629. | Differing risk factor profiles of ischemic stroke subtypes: evidence for a distinct lacunar arteriopathy? |
| 121 | Head & Neck | 2010;32(4): 427-434 | Predition of hypocalcemia after using 1- to 6-hour postoperative parathyroid hormone and calcium levels: an analysis of pooled individual patient data from 3 observational studies. |
| 122 | British Medical Journal | 2011;342: d548. | Association between C reactive protein and coronary heart disease: mendelian randomisation analysis based on individual participant data. |
| 123 | The Lancet Oncology | 2010;11(6): 530-542. | Insulin-like growth factor 1 (IGF1), IGF binding protein 3 (IGFBP3), and breast cancer risk: pooled individual data analysis of 17 prospective studies |
| 124 | Clinical Infectious Diseases | 2010;51(4): 381-389. | Meta-analysis of a possible signal of increased mortality associated with cefepime use. |
| 125 | Journal of Substance Abuse Treatment | 2010;39(3): 218-226. | Effect of early and late compliance on the effectiveness of acamprosate in the treatment of alcohol dependence. |
| 126 | European Journal of Cancer | 2010;46(3): 541-548 | Historical cross-trial comparisons for competing treatments in advanced breast cancer--an empirical analysis of bias |
| 127 | The Lancet Oncology | 2010; 11(9): 835-844. | Long-term outcome in patients with a pathological complete response after chemoradiation for rectal cancer: a pooled analysis of individual patient data. |
| 128 | International Journal of Gynecological Cancer | 2010;20(6): 945-952 | Prognostic relevance of uncommon ovarian histology in women with stage III/IV epithelial ovarian cancer |
| 129 | The Lancet | 2010;375(9731): 2073-2081. | Association of estimated glomerular filtration rate and albuminuria with all-cause and cardiovascular mortality in general population cohorts: a collaborative meta-analysis |
| 130 | Pharmacoepidemiol Drug Safety | 2010;19(2): 196-202. | Pravastatin use and cancer risk: a meta-analysis of individual patient data from long-term prospective controlled trials in Japan |
| 131 | British Medical Journal | 2010;25(341): c6945. | Clinical effectiveness of elective single versus double embryo transfer: meta-analysis of individual patient data from randomised trials. |
| 132 | British Medical Journal | 2010;21(341) | Hysterectomy, endometrial destruction, and levonorgestrel releasing intrauterine system (Mirena) for heavy menstrual bleeding: systematic review and meta-analysis of data from individual patients. |
| 133 | Depression and Anxiety | 2010; 27(1): 12-18. | Anxiety does not predict response to duloxetine in major depression: results of a pooled analysis of individual patient data from 11 placebo-controlled trials. |
| 134 | American Journal of Cardiology | 2010;105(1): 69-76. | Meta-analysis of comparative efficacy of increasing dose of Atorvastatin versus Rosuvastatin versus Simvastatin on lowering levels of atherogenic lipids (from VOYAGER). |
| 135 | Cancer Chemotherapy and Pharmacology | 2010;65(2): 319-324. | Capped-dose mitomycin C: a pooled safety analysis from three prospective clinical trials. |
| 136 | The Journal of the American Medical Association | 2010;303(17): 1729-1737. | Benefit of adjuvant chemotherapy for resectable gastric cancer: a meta-analysis. |
| 137 | Cochrane Database of Systematic Reviews | 2010;(6): CD005225. | Creatine for amyotrophic lateral sclerosis/motor neuron disease. |
| 138 | the Journals of gerontology | 2010;65(3): 258-265. | Red cell distribution width and mortality in older adults: a meta-analysis. |
| 139 | Gastroenterology Clinical Biology | 2010;34(6-7): 388-396. | ActiTest accuracy for the assessment of histological activity grades in patients with chronic hepatitis C, an overview using Obuchowski measure |
| 140 | Journal of Pediatric Hematology/Oncology | 2010;32(6): 454-461. | Efficacy of high-dose chemotherapy and autologous stem cell transplant for recurrent Wilms' tumor: a meta-analysis. |
| 141 | Journal of Nuclear Medicine | 2010;51(10): 1507-1516. | Added value of baseline 18F-FDG uptake in serial 18F-FDG PET for evaluation of response of solid extracerebral tumors to systemic cytotoxic neoadjuvant treatment: a meta-analysis |
| 142 | HIV Clinical Trials | 2010;11(1): 39-50. | A meta-analysis of six placebo-controlled trials of thiazolidinedione therapy for HIV lipoatrophy |
| 143 | Annals of Oncology | 2010;21(2): 312-318. | Efficacy of carboplatin-taxane combinations in the management of castration-resistant prostate cancer: a pooled analysis of seven prospective clinical trials. |
| 144 | The Journal of the American Medical Association | 2010;304(12): 1365-1374. | Subclinical hypothyroidism and the risk of coronary heart disease and mortality. |
| 145 | Ophthalmology | 2010;117(2): 313-319 e311. | The prevalence of retinal vein occlusion: pooled data from population studies from the United States, Europe, Asia, and Australia |
| 146 | Cochrane Database of Systematic Reviews | 2010;(9): CD004332. | Acamprosate for alcohol dependence. |
| 147 | The Lancet | 2010;375(9733): 2215-2222. | Diabetes mellitus, fasting blood glucose concentration, and risk of vascular disease: a collaborative meta-analysis of 102 prospective studies. |
| 148 | EuroIntervention | 2010; 5(7): 788-794. | A pooled gender based analysis comparing the XIENCE V(R) everolimus-eluting stent and the TAXUS paclitaxel-eluting stent in male and female patients with coronary artery disease, results of the SPIRIT II and SPIRIT III studies: two-year analysis. |
| 149 | Current Medical Research & Opinion | 2010;26(4): 925-932. | Sublingual immunotherapy in daily medical practice: effectiveness of different treatment schedules - IPD meta-analysis. |
| 150 | American Journal of Managed Care | 2010;16(9): 688-696. | Preferred roles in treatment decision making among patients with cancer: a pooled analysis of studies using the Control Preferences Scale |
| 151 | Journal of Hepatology | 2010;53(2): 273-282. | Renin-angiotensin-aldosterone inhibitors in the reduction of portal pressure: a systematic review and meta-analysis |
| 152 | International Clinical Psychopharmacology | 2010;25(4): 189-198 | Remission with mirtazapine and selective serotonin reuptake inhibitors: a meta-analysis of individual patient data from 15 controlled trials of acute phase treatment of major depression |
| 153 | British Medical Journal | 2011;25: 777-786. | Liver transplant outcomes in HIV-infected patients: a systematic review and meta-analysis with synthetic cohort |
| 154 | The Lancet | 2010;375(9725): 1536-1544. | Lipoprotein-associated phospholipase A(2) and risk of coronary disease, stroke, and mortality: collaborative analysis of 32 prospective studies |
| 155 | Obstetrics & Gynecology | 2010;116(1): 160-167. | Endometrial Thickness Measurement for Detecting Endometrial Cancer in Women With Postmenopausal Bleeding: A Systematic Review and Meta-Analysis. |
| 156 | The Lancet Neurology | 2010; 9(3): 254-263. | Adjunctive dexamethasone in bacterial meningitis: a meta-analysis of individual patient data. |
| 157 | Annals of Oncology | 2010;21(10): 2023-2028. | The influence of sex and histology on outcomes in non-small-cell lung cancer: a pooled analysis of five randomized trials. |
| 158 | Annals of Oncology | 2010;21(2): 232-237. | The strength of female sex as a prognostic factor in small-cell lung cancer: a pooled analysis of chemotherapy trials from the Manchester Lung Group and Medical Research Council Clinical Trials Unit. |
| 159 | Circulation | 2010; 3(5): 498-505. | Body mass index and risk of stroke and myocardial infarction in a relatively lean population: meta-analysis of 16 Japanese cohorts using individual data |
| 160 | Cochrane Database of Systematic Reviews | 2009;(4): CD004128 | Hypothermia for neuroprotection in adults after cardiopulmonary resuscitation |
| 161 | The Lancet | 2009; 373(9678): 1849-1860. | Aspirin in the primary and secondary prevention of vascular disease: collaborative meta-analysis of individual participant data from randomised trials. |
| 162 | Human Reproduction Update | 1999;5(5):475-482. | Critical analysis of intravenous immunoglobulin therapy for recurrent miscarriage. |
| 163 | American Journal of Sports Medicine | 2009;37(12): 2470-2478. | Patellar Tendon Versus Hamstring Tendon Autografts for Reconstructing the Anterior Cruciate Ligament: A Meta-Analysis Based on Individual Patient Data. |
| 164 | Rheumatology (Oxford) | 2009;48(9): 1122-1127. | Early response to COX-2 inhibitors as a predictor of overall response in osteoarthritis: pooled results from two identical trials comparing etoricoxib, celecoxib and placebo |
| 165 | Cochrane Database of Systematic Reviews | 2009;(3): CD007303. | Erythropoietin or Darbepoetin for patients with cancer--meta-analysis based on individual patient data |
| 166 | Annals of the Rheumatic Diseases | 2009;68(7): 1177-1183 | Etanercept therapy in rheumatoid arthritis and the risk of malignancies: a systematic review and individual patient data meta-analysis of randomised controlled trials |
| 167 | Journal of Clinical Psychiatry | 2001;62:869-77 | Response and remission rates in different subpopulations with major depressive disorder administered venlafaxine, selective serotonin reuptake inhibitors, or placebo |
| 168 | Journal of the American Academy of Dermatology | 2009;61(6): 961-970. | Safety and tolerability of onabotulinumtoxinA in the treatment of facial lines: a meta-analysis of individual patient data from global clinical registration studies in 1678 participants. |
| 169 | European Heart Journal | 2009;30(7): 820-826. | Effect of fish oil on ventricular tachyarrhythmia in three studies in patients with implantable cardioverter defibrillators |
| 170 | European Heart Journal | 2009;30(18): 2193-2203. | Clinical impact of thrombectomy in acute ST-elevation myocardial infarction: an individual patient-data pooled analysis of 11 trials. |
| 171 | American Society for Artificial Internal Organs Journal | 2009;55(6): 581-586. | Use of Extracorporeal Membrane Oxygenation for Adults in Cardiac Arrest (E-CPR): A Meta-Analysis of Observational Studies |
| 172 | Radiology | 2009;251(2): 493-502. | Carotid artery stenosis: accuracy of noninvasive tests--individual patient data meta-analysis. |
| 173 | Annals of Oncology | 2009;20(5): 885-891 | The impact of primary tumour origins in patients with advanced oesophageal, oesophago-gastric junction and gastric adenocarcinoma--individual patient data from 1775 patients in four randomised controlled trials. |
| 174 | British Journal of Haematology | 2009;145(3): 376-388. | Beneficial and harmful effects of anthracyclines in the treatment of childhood acute lymphoblastic leukaemia: a systematic review and meta-analysis |
| 175 | Osteoporosis International | 2009;20(2): 291-297. | Ibandronate for the prevention of nonvertebral fractures: a pooled analysis of individual patient data |
| 176 | Clinical Therapeutics | 2009;31(8): 1641-1651. | Risk for nocturnal hypoglycemia with biphasic insulin aspart 30 compared with biphasic human insulin 30 in adults with type 2 diabetes mellitus: a meta-analysis. |
| 177 | British Medical Journal | 2009;14(338): b603. | Naftidrofuryl for intermittent claudication: meta-analysis based on individual patient data. |
| 178 | Journal of Thrombosis & Thrombolysis | 2009;28(3): 288-298. | Benefits of pharmacological facilitation with glycoprotein IIb-IIIa inhibitors in diabetic patients undergoing primary angioplasty for STEMI. A subanalysis of the EGYPT cooperation |
| 179 | Journal of Vascular Health and Risk Management | 2009; 5(1): 243-247. | Individual patient-data meta-analysis comparing clinical outcome in patients with ST-elevation myocardial infarction treated with percutaneous coronary intervention with or without prior thrombectomy. ATTEMPT study: a pooled Analysis of Trials on ThrombEctomy in acute Myocardial infarction based on individual PatienT data. |
| 180 | Journal of Clinical Oncology | 2009;27(11): 1836-1843. | Meta-analysis of single-agent chemotherapy compared with combination chemotherapy as second-line treatment of advanced non-small-cell lung cancer. |
| 181 | Circulation | 2009;120(20): 2006-2011. | Major bleeding, mortality, and efficacy of fondaparinux in venous thromboembolism prevention trials. |
| 182 | Journal of Human Hypertension | 2009; 23(10): 645-653. | Night-day blood pressure ratio and dipping pattern as predictors of death and cardiovascular events in hypertension |
| 183 | British Journal of Psychiatry | 2009;194(1): 4-9. | Lamotrigine for treatment of bipolar depression: independent meta-analysis and meta-regression of individual patient data from five randomised trials. |
| 184 | Clinical Journal of the American Society of Nephrology | 2009; 4(9): 1449-1458. | Interferon for hepatitis C virus in hemodialysis--an individual patient meta-analysis of factors associated with sustained virological response. |
| 185 | Annals of Emergency Medicine | 2009;54(2): 171-180e174 | Predictors of Emesis and Recovery Agitation With Emergency Department Ketamine Sedation: An Individual-Patient Data Meta-Analysis of 8,282 Children. |
| 186 | Annals of Emergency Medicine | 2009;54(2): 158-168e154. | Predictors of Airway and Respiratory Adverse Events With Ketamine Sedation in the Emergency Department: An Individual-Patient Data Meta-analysis of 8,282 Children. |
| 187 | Neurosurgery | 2009;64(3 Suppl): 145-160. | Operative treatment of anterior thoracic spinal cord herniation: three new cases and an individual patient data meta-analysis of 126 case reports |
| 188 | Primary Care Respiratory Journal | 2009;18(2): 106-113. | Patient-level pooled analysis of the effect of tiotropium on COPD exacerbations and related hospitalisations. |
| 189 | Pharmacotherapy | 2009;29(3): 255-262. | Effect of ropinirole on sleep outcomes in patients with restless legs syndrome: meta-analysis of pooled individual patient data from randomized controlled trials |
| 190 | Liver Transpl | 2009;15(8): 894-906. | Tuberculosis in liver transplant recipients: a systematic review and meta-analysis of individual patient data |
| 191 | Revista Española de Cardiología | 2009;62(4): 354-364. | Drug-eluting stents versus bare-metal stents in diabetic patients with ST-segment elevation acute myocardial infarction: a pooled analysis of individual patient data from seven randomized trials. |
| 192 | Genes & Immunity | 2009;10(5): 495-502. | Factor V Leiden and thrombosis in patients with systemic lupus erythematosus: a meta-analysis |
| 193 | Transfusion | 2009;49(4): 624-635. | Impact of ABO mismatching on the outcomes of allogeneic related and unrelated blood and marrow stem cell transplantations for hematologic malignancies: IPD-based meta-analysis of cohort studies |
| 194 | Injury prevention | 2009;15(3): 197-204. | The effect of education and home safety equipment on childhood thermal injury prevention: meta-analysis and meta-regression. |
| 195 | Journal of Diabetes | 2009;1(3): 173-181. | Smoking, diabetes and cardiovascular diseases in men in the Asia Pacific region |
| 196 | Current Medical Research & Opinion | 2009;25(1): 161-175. | Escitalopram in the treatment of major depressive disorder: a meta-analysis |
| 197 | The Lancet Oncology | 2009;10(8): 772-784. | TP53 codon 72 polymorphism and cervical cancer: a pooled analysis of individual data from 49 studies. |
| 198 | Journal of Clinical Oncology | 2009;27(18): 3000-3006. | Prognostic factors in adult patients up to 60 years old with acute myeloid leukemia and translocations of chromosome band 11q23: individual patient data-based meta-analysis of the German Acute Myeloid Leukemia Intergroup |
| 199 | Journal of Clinical Oncology | 2009;27(17): 2838-2847. | Pooled analysis of individual patient-level data from all randomized, double-blind, placebo-controlled trials of darbepoetin alfa in the treatment of patients with chemotherapy-induced anemia. |
| 200 | Health Technology Assessment | 2009;13(32): 1-207, iii. | Systematic review and individual patient data meta-analysis of diagnosis of heart failure, with modelling of implications of different diagnostic strategies in primary care |
| 201 | American journal of epidemiology | 2009;170(11): 1323-1332. | Prediction of Incident Stroke Events Based on Retinal Vessel Caliber: A Systematic Review and Individual-Participant Meta-Analysis |
| 202 | Annals of Internal Medicine | 2009; 151(6): 404-413. | Meta-analysis: Retinal Vessel Caliber and Risk for Coronary Heart Disease. |
| 203 | British Journal of Anaesthesia | 2009; 103(6): 874-881. | Analysis of individual patient data from clinical trials: epidural morphine for postoperative pain |
| 204 | The Lancet Oncology | 2009;10(4): 341-350. | Surrogate endpoints for overall survival in locally advanced head and neck cancer: meta-analyses of individual patient data |
| 205 | British Medical Journal | 2009;4(338): b1024. | Effectiveness and safety of nicotine replacement therapy assisted reduction to stop smoking: systematic review and meta-analysis. |
| 206 | Clinical Cancer Research | 2009;15(13): 4493-4498 | Combined survival analysis of prospective clinical trials of gefitinib for non-small cell lung cancer with EGFR mutations |
| 207 | Heart | 2009;95(11): 909-916. | Does cigarette smoking exacerbate the effect of total cholesterol and high-density lipoprotein cholesterol on the risk of cardiovascular diseases? |
| 208 | Health Technology Assessment | 2008;12(2):iii-iv, ix-xi, 1-135. | cut down to quit' with nicotine replacement therapies in smoking cessation: a systematic review of effectiveness and economic analysis. |
| 209 | Anticancer Research | 2009;29(7): 2739-2745 | Individual patient based meta-analysis of lentinan for unresectable/recurrent gastric cancer |
| 210 | Annals of Oncology | 2009;20(9): 1529-1534. | Meta-analysis of the REAL-2 and ML17032 trials: evaluating capecitabine-based combination chemotherapy and infused 5-fluorouracil-based combination chemotherapy for the treatment of advanced oesophago-gastric cancer |
| 211 | British Medical Journal | 2009; 18(338). | Four layer bandage compared with short stretch bandage for venous leg ulcers: systematic review and meta-analysis of randomised controlled trials with data from individual patients. |
| 212 | British Medical Journal | 2009; 338(7686): 85-92. | Patients' preferences within randomised trials: systematic review and patient level meta-analysis. |
| 213 | The Lancet Oncology | 2009;10(9): 865-871. | Baseline quality of life as a prognostic indicator of survival: a meta-analysis of individual patient data from EORTC clinical trials. |
| 214 | The Journal of the American Medical Association | 2009;301(23): 2462-2471. | Interaction Between the Serotonin Transporter Gene (5-HTTLPR), Stressful Life Events, and Risk of Depression: A Meta-analysis |
| 215 | Acta Paediatrica | 2009;98(1): 91-97. | IPD meta-analysis shows no effect of LC-PUFA supplementation on infant growth at 18 months |
| 216 | European Journal of Heart Failure | 2009;11(10): 929-936. | Independent relationship of left atrial size and mortality in patients with heart failure: an individual patient meta-analysis of longitudinal data (MeRGE Heart Failure). |
| 217 | Journal of Clinical Oncology | 2009;27(12): 1948-1955. | Pooled safety and efficacy analysis examining the effect of performance status on outcomes in nine first-line treatment trials using individual data from patients with metastatic colorectal cancer. |
| 218 | Bone | 2009;44(3): 423-427. | Ibandronate dose response is associated with increases in bone mineral density and reductions in clinical fractures: results of a meta-analysis. |
| 219 | Cochrane Database of Systematic Reviews | 2009;(1): CD000356. | Early discharge hospital at home. |
| 220 | Canadian Medical Association Journal | 2009;180(2): 175-182. | Avoiding hospital admission through provision of hospital care at home: a systematic review and meta-analysis of individual patient data |
| 221 | The Lancet | 2009;374(9691): 712-719. | Budesonide and the risk of pneumonia: a meta-analysis of individual patient data. |
| 222 | journal of clinical pharmacy and therapeutics | 2009;34(2): 177-186. | Gastrointestinal tolerability of aspirin and the choice of over-the-counter analgesia for short-lasting acute pain |
| 223 | PLOS Neglected Tropical Diseases | 2009;3(9): e524. | Treatment response of cystic echinococcosis to benzimidazoles: a systematic review. |
| 224 | CNS Spectrums | 2009;14(3): 144-154. | An integrated analysis of the efficacy of desvenlafaxine compared with placebo in patients with major depressive disorder |
| 225 | Cochrane Database of Systematic Reviews | 2009;(4): CD007045. | Selective internal radiation therapy for liver metastases from colorectal cancer. |
| 226 | Human Reproduction Update | 2009; 15(1): 5-12 | The clinical significance of the retrieval of a low number of oocytes following mild ovarian stimulation for IVF: a meta-analysis |
| 227 | Acupuncture in Medicine | 2009; 27(3): 126-127. | The Acupuncture Trialists' Collaboration: individual patient data meta-analysis of chronic pain trials (data updated in 2012) |
| 228 | American Journal of Cardiology | 2009;104(11): 1457-1464. | Effects of combination lipid therapy on coronary stenosis progression and clinical cardiovascular events in coronary disease patients with metabolic syndrome: a combined analysis of the Familial Atherosclerosis Treatment Study (FATS), the HDL-Atherosclerosis Treatment Study (HATS), and the Armed Forces Regression Study (AFREGS) |
| 229 | Journal of Clinical Oncology | 2008;26(35): 5802-5812. | Reducing uncertainties about the effects of chemoradiotherapy for cervical cancer: a systematic review and meta-analysis of individual patient data from 18 randomized trials. |
| 230 | Journal of Clinical Oncology | 2008;26(28): 4617-4625. | Chemotherapy in addition to supportive care improves survival in advanced non-small-cell lung cancer: a systematic review and meta-analysis of individual patient data from 16 randomized controlled trials. |
| 231 | European Journal of Heart Failure | 2008;10(8): 786-792. | Independence of restrictive filling pattern and LV ejection fraction with mortality in heart failure: an individual patient meta-analysis. |
| 232 | European Heart Journal | 2008; 29(5): 594-601. | Impact of obesity as a mortality predictor in high-risk patients with myocardial infarction or chronic heart failure: a pooled analysis of five registries |
| 233 | The Journal of the American Medical Association | 2008; 300(2): 197-208. | Ankle Brachial Index Combined With Framingham Risk Score to Predict Cardiovascular Events and Mortality: A Meta-analysis |
| 234 | Human Reproduction | 2008;23(12): 2709-2717. | Reproductive outcome after transplantation of ovarian tissue: a systematic review |
| 235 | The Lancet | 2008;371(9609): 303-314. | Ovarian cancer and oral contraceptives: collaborative reanalysis of data from 45 epidemiological studies including 23,257 women with ovarian cancer and 87,303 controls. |
| 236 | Annals of Surgery | 2008;247(5): 892-898 | Provider and Center Effect in Multicenter Randomized Controlled Trials of Surgical Specialties: An Analysis on Patient-level Data |
| 237 | American journal of epidemiology | 2008;167(5): 505-516. | Meta- and Pooled Analyses of the Methylenetetrahydrofolate Reductase C677T and A1298C Polymorphisms and Gastric Cancer Risk: A Huge-GSEC Review. |
| 238 | Drugs Aging | 2008;25(8):707-714 | Defining treatment response to donepezil in Alzheimer's disease: responder analysis of patient-level data from randomized, placebo-controlled studies |
| 239 | Journal of Clinical Oncology | 2008;26(12): 1987-1992. | Evaluation of tumor response, disease control, progression-free survival, and time to progression as potential surrogate end points in metastatic breast cancer. |
| 240 | Archives of Surgery | 2008;143(1): 75-83. | Influence of Resection Margins and Treatment on Survival in Patients With Pancreatic Cancer: Meta-analysis of Randomized Controlled Trials. |
| 241 | The Lancet | 2008;371(9606): 29-40. | Adjuvant chemotherapy in oestrogen-receptor-poor breast cancer: patient-level meta-analysis of randomised trials |
| 242 | Circulation | 2008;118(11): 1146-1154. | Long-Term Safety and Efficacy of Percutaneous Coronary Intervention With Stenting and Coronary Artery Bypass Surgery for Multivessel Coronary Artery Disease: A Meta-Analysis With 5-Year Patient-Level Data From the ARTS, ERACI-II, MASS-II, and SoS Trials. |
| 243 | Cochrane Database of Systematic Reviews | 2008;(2): CD001368. | Naftidrofuryl for intermittent claudication |
| 244 | Heart | 2008;94(12): 1548-1558. | Early glycoprotein IIb-IIIa inhibitors in primary angioplasty (EGYPT) cooperation: an individual patient data meta-analysis. |
| 245 | Stroke | 2008;39(6): 1751-1758. | NXY-059 for the Treatment of Acute Stroke: Pooled Analysis of the SAINT I and II Trials |
| 246 | Clinical Infectious Diseases | 2008;47(4): 510-516. | Low Serum Mannose-Binding Lectin Level Increases the Risk of Death due to Pneumococcal Infection |
| 247 | Hypertension | 2008;51(1): 55-61. | Daytime and nighttime blood pressure as predictors of death and cause-specific cardiovascular events in hypertension |
| 248 | Haemophilia | 2008;14(5): 903-912. | Immune tolerance with rituximab in congenital haemophilia with inhibitors: a systematic literature review based on individual patients' analysis |
| 249 | Thorax | 1999;54:7-14 | Long term effects of inhaled corticosteroids in chronic obstructive pulmonary disease: a meta-analysis |
| 250 | Cochrane Database of Systematic Reviews | 2008;(1): CD004024. | High-dose chemotherapy with autologous stem cell transplantation in the first line treatment of aggressive non-Hodgkin lymphoma (NHL) in adults |
| 251 | Journal of Neurology, Neurosurgery & Psychiatry | 2008;79(11): 1218-1223. | Dipyridamole plus aspirin versus aspirin alone in secondary prevention after TIA or stroke: a meta-analysis by risk. |
| 252 | Current Medical Research & Opinion | 2008;24(1): 237-245. | Ibandronate and the risk of non-vertebral and clinical fractures in women with postmenopausal osteoporosis: results of a meta-analysis of phase III studies |
| 253 | Epilepsia | 2008;49(2): 343-348. | Severe myoclonic epilepsy in infancy: a systematic review and a meta-analysis of individual patient data |
| 254 | Journal of Clinical Endocrinology & Metabolism | 2008; 93(11): 4245-4253. | Corticotropin Tests for Hypothalamic-Pituitary- Adrenal Insufficiency: A Metaanalysis |
| 255 | Archives of Disease in Childhood | 2008;93(7): 599-608. | Effect of education and safety equipment on poisoning-prevention practices and poisoning: systematic review, meta-analysis and meta-regression |
| 256 | American Journal of Preventive Medicine | 2008;35(4): 370-379. | Preventing childhood falls at home: meta-analysis and meta-regression |
| 257 | Journal of the American College of Cardiology | 2008;51(7): 708-715. | Paclitaxel-eluting coronary stents in patients with diabetes mellitus: pooled analysis from 5 randomized trials |
| 258 | Archives of Otolaryngology—Head & Neck Surgery | 2008;134(2): 128-132. | Antibiotic therapy to prevent the development of asymptomatic middle ear effusion in children with acute otitis media: a meta-analysis of individual patient data. |
| 259 | International Journal of Cancer | 2008;122(1): 144-154 | Personal sun exposure and risk of non Hodgkin lymphoma: a pooled analysis from the Interlymph Consortium |
| 260 | Clinical Biochemistry | 2008;41(16-17): 1368-1376. | Diagnostic accuracy, reproducibility and robustness of fibrosis blood tests in chronic hepatitis C: a meta-analysis with individual data |
| 261 | American Journal of Roentgenology | 2008;191(6): 1667-1675. | Meta-analysis of 40- and 64-MDCT angiography for assessing coronary artery stenosis |
| 262 | American journal of epidemiology | 2008;167(12): 1397-1406. | Alcohol drinking and colorectal cancer in Japanese: a pooled analysis of results from five cohort studies. |
| 263 | Circulation | 2008;117(20): 2591-2598. | Independent prognostic importance of a restrictive left ventricular filling pattern after myocardial infarction: an individual patient meta-analysis: Meta-Analysis Research Group in Echocardiography acute myocardial infarction. |
| 264 | Journal of Psychiatric Research | 2008;42(2): 134-140. | Efficacy of bupropion and the selective serotonin reuptake inhibitors in the treatment of anxiety symptoms in major depressive disorder: a meta-analysis of individual patient data from 10 double-blind, randomized clinical trials. |
| 265 | Critical Care Medicine | 2008;36(4): 1323-1329. | Effect of dopexamine infusion on mortality following major surgery: Individual patient data meta-regression analysis of published clinical trials |
| 266 | Journal of Clinical Oncology | 2008;26(12): 1980-1986. | Taxanes alone or in combination with anthracyclines as first-line therapy of patients with metastatic breast cancer. |
| 267 | Journal of the American College of Cardiology | 2008;51(1): 23-32. | Angiographic surrogate end points in drug-eluting stent trials: a systematic evaluation based on individual patient data from 11 randomized, controlled trials |
| 268 | Journal of Psychiatric Research | 2008; 42(12): 1042-1049. | Examining quality of life in patients with generalized anxiety disorder: clinical relevance and response to duloxetine treatment |
| 269 | Journal of the National Cancer Institute | 2008;100(3): 170-183. | Endogenous sex hormones and prostate cancer: a collaborative analysis of 18 prospective studies. |
| 270 | Annals of Internal Medicine | 2008;149(7): 461-471. | Insulin-like growth factors, their binding proteins, and prostate cancer risk: analysis of individual patient data from 12 prospective studies. |
| 271 | Cochrane Database of Systematic Reviews | 2008;(4): CD007491. | Admission avoidance hospital at home. |
| 272 | Circulation | 2008;117(16): 2104-2113. | Cardiovascular risk of celecoxib in 6 randomized placebo-controlled trials: the cross trial safety analysis. |
| 273 | Nephrology Dialysis Transplantation | 2008;23(9): 3017-3023. | Association between cytokine gene polymorphisms and outcomes in renal transplantation: a meta-analysis of individual patient data |
| 274 | Journal of Acquired Immune Deficiency Syndromes | 2008;49(5): 523-531. | Low Risk of Death, but Substantial Program Attrition, in Pediatric HIV Treatment Cohorts in Sub-Saharan Africa. |
| 275 | British Journal of Cancer | 2008;98(2): 294-299. | Oxygen for relief of dyspnoea in mildly- or non-hypoxaemic patients with cancer: a systematic review and meta-analysis. |
| 276 | Circulation | 2008; 1(1): 43-50. | Mutations in the HFE Gene and Cardiovascular Disease Risk: An Individual Patient Data Meta-Analysis of 53 880 Subjects. |
| 277 | Journal of Vascular Surgery | 2008;47(6): 1364-1370e1361 | Suprarenal endograft fixation and medium-term renal function: Systematic review and meta-analysis |
| 278 | The Lancet | 2008; 371(9616): 908-914. | Antibiotics for adults with clinically diagnosed acute rhinosinusitis: a meta-analysis of individual patient data |
| 279 | International Journal of Cancer | 2006;120(4): 885-891. | Comparison of risk factors for invasive squamous cell carcinoma and adenocarcinoma of the cervix: collaborative reanalysis of individual data on 8,097 women with squamous cell carcinoma and 1,374 women with adenocarcinoma from 12 epidemiological studies. |
| 280 | Journal of the National Cancer Institute | 2007;99(11): 847-857. | Cisplatin- Versus Carboplatin-Based Chemotherapy in First-Line Treatment of Advanced Non-Small-Cell Lung Cancer: An Individual Patient Data Meta-analysis. |
| 281 | The Lancet | 2007;369(9575): 1791-1798. | Antiplatelet agents for prevention of pre-eclampsia: a meta-analysis of individual patient data. |
| 282 | European Journal of Cardiovascular Prevention & Rehabilitation | 2007;14(1): 3-11. | Collaborative meta-analysis of individual participant data from observational studies of Lp-PLA2 and cardiovascular diseases |
| 283 | Rheumatology (Oxford) | 2007;46(10): 1601-1605. | Conservative treatments for tennis elbow do subgroups of patients respond differently |
| 284 | Journal of Thoracic Oncology | 2007 2(8): S366-S367. | Chemotherapy (CT) in addition to surgery or surgery plus radiotherapy (RT) in non-small cell lung cancer (NSCLC): Two meta-analyses using individual patient data (IPD) from randomised controlled trials (RCTs) |
| 285 | Journal of Thoracic Oncology | 2007; 2(8): S337. | Supportive care and chemotherapy (CT) versus supportive care alone in advanced non-small cell lung cancer (NSCLC): A meta-analysis using individual patient data (IPD) from randomised controlled trials (RCTs): B2-03. |
| 286 | Epilepsia | 2007;48(6): 1173-1178. | New statistical method for analyzing time to first seizure: example using data comparing carbamazepine and valproate monotherapy |
| 287 | The Lancet | 2007;369(9574): 1711-1723. | Use of luteinising-hormone-releasing hormone agonists as adjuvant treatment in premenopausal patients with hormone-receptor-positive breast cancer: a meta-analysis of individual patient data from randomised adjuvant trials |
| 288 | European Journal of Epidemiology | 2007;22(12): 839-869. | The Emerging Risk Factors Collaboration: analysis of individual data on lipid, inflammatory and other markers in over 1.1 million participants in 104 prospective studies of cardiovascular diseases |
| 289 | Age Ageing | 2007; 36(2): 219-222. | The effect of exercise on outcomes for hospitalised older acute medical patients: an individual patient data meta-analysis. |
| 290 | Journal of Clinical Oncology | 2007;25(11): 1377-1382. | Individual patient data meta-analysis of docetaxel administered once every 3 weeks compared with once every week second-line treatment of advanced non-small-cell lung cancer. |
| 291 | Journal of palliative care | 2007;23(4): 245-252; | Meta-analysis of survival prediction with Palliative Performance Scale |
| 292 | The Lancet Oncology | 2007; 8(11): 994-1000. | Addition of estramustine to chemotherapy and survival of patients with castration-refractory prostate cancer: a meta-analysis of individual patient data |
| 293 | British Journal of Nutrition | 2007;98(1): 17-25. | Associations between postprandial insulin and blood glucose responses, appetite sensations and energy intake in normal weight and overweight individuals: a meta-analysis of test meal studies |
| 294 | BJOG: An International Journal of Obstetrics & Gynaecology | 2007;114(12): 1460-1476. | Cervical stitch (cerclage) for preventing pregnancy loss: individual patient data meta-analysis. |
| 295 | American journal of epidemiology | 2007;166(8): 867-879. | Associations of plasma fibrinogen levels with established cardiovascular disease risk factors, inflammatory markers, and other characteristics: individual participant meta-analysis of 154,211 adults in 31 prospective studies: the fibrinogen studies collaboration. |
| 296 | European Heart Journal | 2007;28(22): 2706-2713. | Meta-analysis of randomized trials on drug-eluting stents vs. bare-metal stents in patients with acute myocardial infarction. |
| 297 | New England Journal of Medicine | 2007;356(10): 1030-1039. | Analysis of 14 trials comparing sirolimus-eluting stents with bare-metal stents. |
| 298 | Cochrane Database of Systematic Reviews | 2007;(1): CD005014. | Home safety education and provision of safety equipment for injury prevention. |
| 299 | Journal of Neurology | 2007;254(6): 705-712. | Efficacy and safety of 1,000 mg effervescent aspirin: individual patient data meta-analysis of three trials in migraine headache and migraine accompanying symptoms |
| 300 | Neurology | 2007;69(8): 766-775. | Meta-analysis of APOE genotype and subarachnoid hemorrhage: clinical outcome and delayed ischemia |
| 301 | The Lancet | 2007;370(9602): 1829-1839. | Blood cholesterol and vascular mortality by age, sex, and blood pressure: a meta-analysis of individual data from 61 prospective studies with 55,000 vascular deaths |
| 302 | The Journal of the American Medical Association | 2007; 298(10): 1180-1188. | Pioglitazone and risk of cardiovascular events in patients with type 2 diabetes mellitus: a meta-analysis of randomized trials |
| 303 | Arthritis & Rheumatism | 2007; 56(8): 2789-2797. | Adjunctive methotrexate for treatment of giant cell arteritis: an individual patient data meta-analysis |
| 304 | European Heart Journal | 2007;28(4): 443-449. | Abciximab in primary coronary stenting of ST-elevation myocardial infarction: a European meta-analysis on individual patients' data with long-term follow-up. |
| 305 | Clinical Therapeutics | 2007;29(8): 1607-1619. | Negative binomial meta-regression analysis of combined glycosylated hemoglobin and hypoglycemia outcomes across eleven Phase III and IV studies of insulin glargine compared with neutral protamine Hagedorn insulin in type 1 and type 2 diabetes mellitus. |
| 306 | Journal of the American College of Surgeons | 2007;205(6): 748-754. | Early prediction of hypocalcemia after thyroidectomy using parathyroid hormone: an analysis of pooled individual patient data from nine observational studies." |
| 307 | Journal of Clinical Psychiatry | 2007;68(12): 1907-1912. | Comparing the rapidity of response during treatment of major depressive disorder with bupropion and the SSRIs: a pooled survival analysis of 7 double-blind, randomized clinical trials |
| 308 | European Archives of Oto-Rhino-Laryngology | 2007;264(6): 587-594. | Activity of chemotherapy in the palliative treatment of salivary gland tumors: review of the literature. |
| 309 | Pediatrics | 2007;119(3): 579-585. | Predictors of pain and/or fever at 3 to 7 days for children with acute otitis media not treated initially with antibiotics: a meta-analysis of individual patient data |
| 310 | British Journal of Cancer | 2007; 96(8): 1170-1177. | An individual patient data meta-analysis of adjuvant therapy with uracil-tegafur (UFT) in patients with curatively resected rectal cancer |
| 311 | Gastroenterology | 2007;133(3): 825-834. | Transjugular intrahepatic portosystemic shunt for refractory ascites: a meta-analysis of individual patient data. |
| 312 | Haematologica | 2007;92(6): 763-770. | Prognosis of acute myeloid leukemia patients up to 60 years of age exhibiting trisomy 8 within a non-complex karyotype: individual patient data-based meta-analysis of the German Acute Myeloid Leukemia Intergroup |
| 313 | British Journal of Urology International | 2007;99(5): 1056-1065. | International study into the use of intermittent hormone therapy in the treatment of carcinoma of the prostate: a meta-analysis of 1446 patients |
| 314 | Annals of Internal Medicine | 2007;147(1): 10-18. | Beta-blockers and progression of coronary atherosclerosis: pooled analysis of 4 intravascular ultrasonography trials. |
| 315 | Chest | 2007; 131(3): 682-689. | A pooled analysis of FEV1 decline in COPD patients randomized to inhaled corticosteroids or placebo. |
| 316 | New England Journal of Medicine | 2007;356(10): 989-997. | A pooled analysis of data comparing sirolimus-eluting stents with bare-metal stents. |
| 317 | Circulation | 2007;115(22): 2842-2847. | Offsetting impact of thrombosis and restenosis on the occurrence of death and myocardial infarction after paclitaxel-eluting and bare metal stent implantation |
| 318 | Journal of Clinical Psychopharmacology | 2007;27(6): 672-676. | Efficacy of duloxetine and selective serotonin reuptake inhibitors: comparisons as assessed by remission rates in patients with major depressive disorder. |
| 319 | Stroke | 2007; 38(6): 1911-1915. | Can We Improve the Statistical Analysis of Stroke Trials?: Statistical Reanalysis of Functional Outcomes in Stroke Trials. |
| 320 | The Lancet | 2007;369:115-22 | Effectiveness of Prenatal Treatment for Congenital Toxoplasmosis: A Meta-analysis of Individual Patients' Data |
| 321 | Archives of Internal Medicine | 2007;167(13): 1353-1359 | Primary Percutaneous Coronary Intervention Compared With Fibrinolysis for Myocardial Infarction in Diabetes Mellitus: Results From the Primary Coronary Angioplasty vs Thrombolysis-2 Trial. |
| 322 | The Lancet Oncology | 2007;8(6): 488-499. | Microvessel density as a prognostic factor in non-small-cell lung carcinoma: a meta-analysis of individual patient data. |
| 323 | Journal of Thoracic Oncology | 2007; 2(5): 430-439. | Epidermal Growth Factor Receptor Mutations and Their Correlation with Gefitinib Therapy in Patients with Non-small Cell Lung Cancer: A Meta-Analysis Based on Updated Individual Patient Data from Six Medical Centers in Mainland China. |
| 324 | Cochrane Database of Systematic Reviews | 2006;(2):CD006018. | Adjuvant chemotherapy for invasive bladder cancer (individual patient data). |
| 325 | International Journal of Cancer | 2006;119(5): 1108-1124. | Cervical carcinoma and reproductive factors: collaborative reanalysis of individual data on 16,563 women with cervical carcinoma and 33,542 women without cervical carcinoma from 25 epidemiological studies." |
| 326 | European Journal of Cardiovascular Prevention & Rehabilitation | 2006;13(1): 30-36. | Coronary risk prediction for those with and without diabetes |
| 327 | European Journal of Heart Failure | 2006;8(1): 90-96 | Effect of angiotensin-converting enzyme inhibition on functional class in patients with left ventricular systolic dysfunction--a meta-analysis |
| 328 | The Annals of Thoracic Surgery | 2006;81(4): 1529-1535. | Physiologic versus anatomic repair of congenitally corrected transposition of the great arteries: meta-analysis of individual patient data |
| 329 | International Journal of Cancer | 2006; 118(6): 1481-1495. | Carcinoma of the cervix and tobacco smoking: collaborative reanalysis of individual data on 13,541 women with carcinoma of the cervix and 23,017 women without carcinoma of the cervix from 23 epidemiological studies. |
| 330 | Annals of Oncology | 2006;17(3): 473-483. | Concomitant radio-chemotherapy based on platin compounds in patients with locally advanced non-small cell lung cancer (NSCLC): A meta-analysis of individual data from 1764 patients. |
| 331 | Cochrane Database of Systematic Reviews | 2006;(4): CD004329. | Chemotherapy as an adjunct to radiotherapy in locally advanced nasopharyngeal carcinoma. |
| 332 | International Journal of Radiation Oncology*Biology*Physics | 2006;64(1): 47-56. | Chemotherapy in locally advanced nasopharyngeal carcinoma: an individual patient data meta-analysis of eight randomized trials and 1753 patients. |
| 333 | Bone Marrow Transplant | 2006;38(8): 539-546. | Individual patient data meta-analysis of allogeneic peripheral blood stem cell transplant vs bone marrow transplant in the management of hematological malignancies: indirect assessment of the effect of day 11 methotrexate administration. |
| 334 | The Lancet | 2006;368(9538): 843-854. | Hyperfractionated or accelerated radiotherapy in head and neck cancer: a meta-analysis. |
| 335 | Cephalalgia | 2006;26(4): 400-408. | Efficacy, speed of action and tolerability of almotriptan in the acute treatment of migraine: pooled individual patient data from four randomized, double-blind, placebo-controlled clinical trials. |
| 336 | Scandinavian Journal of Work, Environment & Health | 2006;32 Suppl 1: 1-83. | Residential radon and lung cancer--detailed results of a collaborative analysis of individual data on 7148 persons with lung cancer and 14,208 persons without lung cancer from 13 epidemiologic studies in Europe |
| 337 | The American Journal of Surgery | 2006; 191(6): 773-778. | How accurate are published recurrence rates after rectal prolapse surgery? A meta-analysis of individual patient data |
| 338 | Annals of Oncology | 2006;17(12): 1749-1760. | Second malignancy risk associated with treatment of Hodgkin's lymphoma: meta-analysis of the randomised trials. |
| 339 | Cancer Causes Control | 1999;10(2):157-66 | A pooled analysis of case-control studies of thyroid cancer. III. Oral contraceptives, menopausal replacement therapy and other female hormones |
| 340 | Neurology | 2006;66(9): 1310-1317. | A meta-analysis of individual patient responses to lamotrigine or carbamazepine monotherapy. |
| 341 | Journal of the National Cancer Institute | 2006;98(1): 26-38. | Survival effects of postmastectomy adjuvant radiation therapy using biologically equivalent doses: a clinical perspective |
| 342 | Circulation | 2006;114(1): 11-17. | Cardiovascular Outcomes With Atrial-Based Pacing Compared With Ventricular Pacing: Meta-Analysis of Randomized Trials, Using Individual Patient Data |
| 343 | Neuromodulation | 2006; 9(4): 253-261 | Deep Brain Stimulation for Dystonia: A Meta-Analysis |
| 344 | Diabetes | 2006;55(10): 2915-2921. | IL6 Gene Promoter Polymorphisms and Type 2 Diabetes: Joint Analysis of Individual Participants' Data From 21 Studies. |
| 345 | Cochrane Database of Systematic Reviews | 2002;(4):CD002197. | Open mesh versus non-mesh for repair of femoral and inguinal hernia |
| 346 | American Journal of Obstetrics & Gynecology | 2006;194(3): 760-767. | Exercise and bone mineral density at the femoral neck in postmenopausal women: a meta-analysis of controlled clinical trials with individual patient data. |
| 347 | Gastroenterology | 2006;130(6): 1636-1642. | Relationship between steatosis, inflammation, and fibrosis in chronic hepatitis C: a meta-analysis of individual patient data. |
| 348 | journal of clinical pharmacy and therapeutics | 2006;31(2): 161-165. | How high should total pain-relief score be to obviate the need for analgesic remedication in acute pain? Estimation using signal detection theory and individual-patient meta-analysis. |
| 349 | The American Journal of Tropical Medicine and Hygiene | 2006;74(6): 991-998. | Efficacy and safety of the six-dose regimen of artemether-lumefantrine in pediatrics with uncomplicated Plasmodium falciparum malaria: a pooled analysis of individual patient data |
| 350 | Journal of the American College of Cardiology | 2006;48(4): 692-699. | A meta-analysis of the renal safety of isosmolar iodixanol compared with low-osmolar contrast media. |
| 351 | Acta Tropica | 2006;100(1-2): 41-53. | Efficacy and safety of the six-dose regimen of artemether-lumefantrine for treatment of uncomplicated Plasmodium falciparum malaria in adolescents and adults: a pooled analysis of individual patient data from randomized clinical trials |
| 352 | Journal of Thrombosis & Haemostasis | 2007;97(6):931-7 | Rivaroxaban for thromboprophylaxis after orthopaedic surgery: pooled analysis of two studies |
| 353 | The Lancet | 2006;368(9545): 1429-1435. | Antibiotics for acute otitis media: a meta-analysis with individual patient data. |
| 354 | Clinical Therapeutics | 2007;29(9):1950-1956 | Pooled analysis of two clinical trials comparing the clinical outcomes of topical ciprofloxacin/dexamethasone otic suspension and polymyxin B/neomycin/hydrocortisone otic suspension for the treatment of acute otitis externa in adults and children |
| 355 | Osteoarthritis Cartilage | 2006;14(9): 859-866. | An integrated analysis of five double-blind, randomized controlled trials evaluating the safety and efficacy of a hyaluronan product for intra-articular injection in osteoarthritis of the knee. |
| 356 | Journal of Acquired Immune Deficiency Syndromes | 2005;39(4):430-8. | Mortality among HIV-1-infected women according to children's feeding modality: an individual patient data meta-analysis |
| 357 | European Urology | 2005;48(2): 189-201. | Adjuvant chemotherapy in invasive bladder cancer: a systematic review and meta-analysis of individual patient data Advanced Bladder Cancer (ABC) Meta-analysis Collaboration |
| 358 | The Lancet | 2005;365(9472): 1687-1717. | Effects of chemotherapy and hormonal therapy for early breast cancer on recurrence and 15-year survival: an overview of the randomised trials |
| 359 | Cochrane Database of Systematic Reviews | 2005;(2): CD005246. | Neo-adjuvant chemotherapy for invasive bladder cancer |
| 360 | Cochrane Database of Systematic Reviews | 2005;(2): CD000443. | Services for reducing duration of hospital care for acute stroke patients |
| 361 | Obstetrics & Gynecology | 2005;106(1): 181-189. | Cerclage for Short Cervix on Ultrasonography: Meta-Analysis of Trials Using Individual Patient-Level Data. |
| 362 | Circulation | 2005;111(3): 278-287 | Cholesteryl ester transfer protein TaqIB variant, high-density lipoprotein cholesterol levels, cardiovascular risk, and efficacy of pravastatin treatment: individual patient meta-analysis of 13,677 subjects |
| 363 | New England Journal of Medicine | 2005;353(8): 793-802. | A pooled analysis of bone marrow micrometastasis in breast cancer. |
| 364 | Journal of Clinical Oncology | 2005;23(22): 5117-5125. | Objective response to chemotherapy as a potential surrogate end point of survival in metastatic breast cancer patients. |
| 365 | Lung Cancer | 2005;47(1): 81-83 | Postoperative radiotherapy in non-small-cell lung cancer: update of an individual patient data meta-analysis |
| 366 | Oncologist | 2005;10(1): 63-71. | Prior red blood cell transfusions in cancer patients increase the risk of subsequent transfusions with or without recombinant human erythropoietin management. |
| 367 | Osteoporosis International | 2005;16(11): 1330-1338. | Body mass index as a predictor of fracture risk: a meta-analysis. |
| 368 | The Clinical Journal of Pain | 2005;21(3): 241-250. | The time to onset and overall analgesic efficacy of rofecoxib 50 mg: a meta-analysis of 13 randomized clinical trials. |
| 369 | Aging Health | 2005;1(1): 19-26. | Modified-release dipyridamole combined with aspirin for secondary stroke prevention. |
| 370 | Stroke | 2005;36(7): 1360-1365. | Smoking and elevated blood pressure are the most important risk factors for subarachnoid hemorrhage in the Asia-Pacific region: an overview of 26 cohorts involving 306,620 participants. |
| 371 | The Journal of the American Medical Association | 2005;294(14): 1799-1809. | Plasma Fibrinogen Level and the Risk of Major Cardiovascular Diseases and Nonvascular Mortality: An Individual Participant Meta-analysis |
| 372 | Gastroenterology | 2005;128(7): 1838-1844 | Helicobacter pylori "test and treat" or endoscopy for managing dyspepsia: an individual patient data meta-analysis. |
| 373 | Cochrane Database of Systematic Reviews | 2005;(4): CD003187. | Chemotherapy, radiotherapy and combined modality for Hodgkin's disease, with emphasis on second cancer risk. |
| 374 | Digestive Diseases & Sciences | 2005;50(4): 609-616. | Meta-analysis of Outcome of Cytomegalovirus Colitis in Immunocompetent Hosts. |
| 375 | Critical Care Medicine | 2005;33(2): 414-418. | Hypothermia for neuroprotection after cardiac arrest: Systematic review and individual patient data meta-analysis |
| 376 | Journal of Women's Health | 2005;14(9): 820-828. | Does sex matter in the associations between classic risk factors and fatal coronary heart disease in populations from the Asia-Pacific region? http://online.liebertpub.com/doi/pdfplus/10.1089/jwh.2005.14.820 |
| 377 | American Journal of Medicine | 2005; 118(1): 2-10. | Mortality in systemic sclerosis: an international meta-analysis of individual patient data. |
| 378 | Stroke | 2005;36(1): 162-168 | Dipyridamole for Preventing Recurrent Ischemic Stroke and Other Vascular Events: A Meta-Analysis of Individual Patient Data From Randomized Controlled Trials. |
| 379 | Biological Psychiatry | 2005;57(12): 1543-1549. | Early-onset hypothesis of antipsychotic drug action: a hypothesis tested, confirmed and extended. |
| 380 | Medicine (Baltimore) | 2005;84(4): 250-259. | A Meta-Analysis on Data From 575 Patients With Multiple Myeloma Randomly Assigned to Either High-Dose Therapy or Conventional Therapy |
| 381 | Journal of Thoracic & Cardiovascular Surgery | 2005;130(2): 512-519. | One-year outcomes of coronary artery bypass graft surgery versus percutaneous coronary intervention with multiple stenting for multisystem disease: A meta-analysis of individual patient data from randomized clinical trials. |
| 382 | Chest | 2005;128(4): 2203-2210. | Enoxaparin in the Treatment of Deep Vein Thrombosis With or Without Pulmonary Embolism*: An Individual Patient Data Meta-analysis |
| 383 | Pain | 2005;116(3): 322-331. | Acute pain: individual patient meta-analysis shows the impact of different ways of analysing and presenting results. |
| 384 | Journal of Internal Medicine | 1999;14:718-29 | Efficacy of treating hypertension in women |
| 385 | Nephrology Dialysis Transplantation | 2005;20(8): 1617-1622. | Measured creatinine clearance from timed urine collections substantially overestimates glomerular filtration rate in patients with liver cirrhosis: a systematic review and individual patient meta-analysis |
| 386 | Journal of Clinical Oncology | 2005;23(10): 2215-2223. | Meta-analysis to evaluate the role of interferon in follicular lymphoma. |
| 387 | Depression and Anxiety | 2008;25(7):E1-11 | Efficacy of duloxetine in the treatment of generalized anxiety disorder in patients with clinically significant pain symptoms |
| 388 | Archives of Disease in Childhood | 2005;90(5): 480-485. | Grommets in otitis media with effusion: an individual patient data meta-analysis. |
| 389 | Japanese Journal of Clinical Oncology | 2005;35(9): 536-544. | An individual patient data meta-analysis of adjuvant therapy with carmofur in patients with curatively resected colon cancer |
| 390 | Thorax | 2005;60(12): 992-997. | Inhaled corticosteroids and mortality in chronic obstructive pulmonary disease. |
| 391 | Journal of Clinical Oncology | 2005;23(22):5074-5087. | Allogeneic Peripheral Blood Stem-Cell Compared With Bone Marrow Transplantation in the Management of Hematologic Malignancies: An Individual Patient Data Meta-Analysis of Nine Randomized Trials. |
| 392 | Cochrane Database of Systematic Reviews | 2005;(2): CD004446. | Emergency ultrasound-based algorithms for diagnosing blunt abdominal trauma. |
| 393 | British Journal of Cancer | 2005;92(8): 1372-1381. | Meta-analysis of randomised adjuvant therapy trials for pancreatic cancer. |
| 394 | Journal of Clinical Psychiatry | 2005;66(8): 974-981. | Remission rates following antidepressant therapy with bupropion or selective serotonin reuptake inhibitors: a meta-analysis of original data from 7 randomized controlled trials |
| 395 | International Journal of Cancer | 2005;115(5): 799-806. | Parental occupation and Ewing's sarcoma: pooled and meta-analysis |
| 396 | Alimentary Pharmacology & Therapeutics | 2005;21(5): 539-547 | Dynamic decision analysis to determine optimal treatment duration in chronic hepatitis C |
| 397 | Hypertension | 2005;45(2): 203-208. | Short- and long-term incidence of stroke in white-coat hypertension. |
| 398 | Journal of Bone and Mineral Research | 2005;20(12): 2097-2104. | Relationship between changes in BMD and nonvertebral fracture incidence associated with risedronate: reduction in risk of nonvertebral fracture is not related to change in BMD |
| 399 | European Journal of Cardiovascular Prevention & Rehabilitation | 2005;12(5): 484-491. | A comparison of the associations between risk factors and cardiovascular disease in Asia and Australasia |
| 400 | European Heart Journal | 2005;26(14): 1385-1393. | Temporal trends on the risk of arrhythmic vs. non-arrhythmic deaths in high-risk patients after myocardial infarction: a combined analysis from multicentre trials |
| 401 | Cochrane Database of Systematic Reviews | 2004;(2): CD001774. | Neoadjuvant chemotherapy for locally advanced cervix cancer. |
| 402 | Hepatology | 2004;39(2): 333-342. | Effect of peginterferon alfa-2a on liver histology in chronic hepatitis C: a meta-analysis of individual patient data |
| 403 | Journal of Clinical Oncology | 2004;22(12): 2395-2403. | Multivariate prognostic factor analysis in locally advanced and metastatic esophago-gastric cancer--pooled analysis from three multicenter, randomized, controlled trials using individual patient data. |
| 404 | British Medical Journal | 2004;328(7433): 189. | Exercise training meta-analysis of trials in patients with chronic heart failure (ExTraMATCH) |
| 405 | Journal of Neurology, Neurosurgery & Psychiatry | 2004;75(11): 1547-1551 | Does reducing spasticity translate into functional benefit? An exploratory meta-analysis |
| 406 | Circulation | 2004;110(16): 2287-2292. | Selecting patients with atrial fibrillation for anticoagulation: stroke risk stratification in patients taking aspirin |
| 407 | Arthritis & Rheumatism | 2004;50(3): 753-762. | Lack of association of the HLA-DRB1 shared epitope with rheumatoid nodules: an individual patient data meta-analysis of 3,272 Caucasian patients with rheumatoid arthritis |
| 408 | Arthritis & Rheumatism | 2004;50(11): 3476-3484. | Particular HLA-DRB1 Shared Epitope Genotypes Are Strongly Associated With Rheumatoid Vasculitis |
| 409 | Arthritis & Rheumatism | 2004;50(2): 400-412. | Impact of Shared Epitope Genotype and Ethnicity on Erosive Disease: A Meta-Analysis of 3,240 Rheumatoid Arthritis Patients |
| 410 | Journal of Women's Health | 2004;13(3): 293-300. | Efficacy of resistance exercise on lumbar spine and femoral neck bone mineral density in premenopausal women: a meta-analysis of individual patient data |
| 411 | Journal of Hepatology | 2004;40(3): 478-483. | Combination therapy with amantadine and interferon in naive patients with chronic hepatitis C: meta-analysis of individual patient data from six clinical trials |
| 412 | Annals of Oncology | 2004;15(7): 1013-1017 | Predictive factors of survival in patients with advanced colorectal cancer: an individual data analysis of 602 patients included in irinotecan phase III trials |
| 413 | European Neuropsychopharmacology | 2001;11(2):173-180. | Short-term efficacy of tricyclic antidepressants revisited: a meta-analytic study. |
| 414 | Circulation | 2004;110(17): 2678-2686. | Serum triglycerides as a risk factor for cardiovascular diseases in the Asia-Pacific region. |
| 415 | Journal of Infectious Diseases | 2004;189(12): 2154-2166 | Late Postnatal Transmission of HIV-1 in Breast-Fed Children: An Individual Patient Data Meta-Analysis. |
| 416 | Journal of Clinical Oncology | 2004;22(3): 484-492. | Efficacy of oral adjuvant therapy after resection of colorectal cancer: 5-year results from three randomized trials. |
| 417 | Antiviral Therapy | 2004;9(2): 275-286. | Meta-analysis of mutations in the NS5A gene and hepatitis C virus resistance to interferon therapy: uniting discordant conclusions. |
| 418 | Journal of Clinical Oncology | 2004;22(18): 3741-3750. | Individual patient data-based meta-analysis of patients aged 16 to 60 years with core binding factor acute myeloid leukemia: a survey of the German Acute Myeloid Leukemia Intergroup |
| 419 | Anesthesiology | 2004;100(1): 142-148. | Labor analgesia and cesarean delivery: an individual patient meta-analysis of nulliparous women |
| 420 | Journal of Clinical Oncology | 2004;22(18): 3766-3775. | Modulation of Fluorouracil by Leucovorin in Patients With Advanced Colorectal Cancer: An Updated Meta-Analysis. |
| 421 | Cancer Treat Report | 1987;71:7-14 | Overview of randomized trials comparing radical mastectomy without radiotherapy against simple mastectomy with radiotherapy in breast cancer |
| 422 | Gut | 200453(10): 1504-1508. | Long term clinical outcome of chronic hepatitis C patients with sustained virological response to interferon monotherapy. |
| 423 | Stroke | 2004;35(9): 2226-2232. | Individual Patient Data Meta-Analysis of Randomized Controlled Trials of Community Occupational Therapy for Stroke Patients. |
| 424 | International Journal of Geriatric Psychiatry | 2004;19(7): 624-633. | Donepezil for the symptomatic treatment of patients with mild to moderate Alzheimer's disease: a meta-analysis of individual patient data from randomised controlled trials. |
| 425 | European Journal of Cancer | 2003;39(17): 2470-2486. | Neoadjuvant chemotherapy for locally advanced cervical cancer: a systematic review and meta-analysis of individual patient data from 21 randomised trials. |
| 426 | Cochrane Database of Systematic Reviews | 2005;(1): CD002142 | Postoperative radiotherapy for non-small cell lung cancer |
| 427 | Cochrane Database of Systematic Reviews | 2003;(1): CD000442. | Selegiline for Alzheimer's disease |
| 428 | European Journal of Heart Failure | 2003;5(3): 281-289. | Beta-blocker benefit according to severity of heart failure. |
| 429 | British Medical Journal | 2003;326(7401): 1247-1250. | Meta-analysis of data on costs from trials of counselling in primary care: using individual patient data to overcome sample size limitations in economic analyses |
| 430 | Alimentary Pharmacology & Therapeutics | 2003;17(1): 99-109. | Risk factors for failure of Helicobacter pylori therapy--results of an individual data analysis of 2751 patients |
| 431 | Journal of Clinical Oncology | 2003;21(9): 1798-1809. | CNS-directed therapy for childhood acute lymphoblastic leukemia: Childhood ALL Collaborative Group overview of 43 randomized trials |
| 432 | Journal of Periodontology | 2003;74(5): 741-756. | Evidence-based mucogingival therapy. Part 2: Ordinary and individual patient data meta-analyses of surgical treatment of recession using complete root coverage as the outcome variable http://www.joponline.org/doi/pdfplus/10.1902/jop.2003.74.5.741 |
| 433 | American Heart Journal | 2003;145(1): 47-57. | Primary coronary angioplasty compared with intravenous thrombolytic therapy for acute myocardial infarction: Six-month follow up and analysis of individual patient data from randomized trials. |
| 434 | Brain | 2003;126(9): 1940-1954. | Prognosis of vertebrobasilar transient ischaemic attack and minor stroke |
| 435 | Nephrology Dialysis Transplantation | 2003; 18(10): 2047-2053. | The rate of progression of renal disease may not be slower in women compared with men: a patient-level meta-analysis |
| 436 | Annals of Internal Medicine | 2003;139(4): 244-252. | Progression of chronic kidney disease: the role of blood pressure control, proteinuria, and angiotensin-converting enzyme inhibition: a patient-level meta-analysis |
| 437 | Epilepsia | 2003;44(10): 1350-1352. | Seizure-free days observed in randomized placebo-controlled add-on trials with levetiracetam in partial epilepsy |
| 438 | Epilepsy Research | 2005;64(1-2):1-11. | Effect of levetiracetam on the pharmacokinetics of adjunctive antiepileptic drugs: a pooled analysis of data from randomized clinical trials |
| 439 | Cochrane Database of Systematic Reviews | 2003;(1): CD001785. | Laparoscopic techniques versus open techniques for inguinal hernia repair. |
| 440 | European Journal of Anaesthesiology | 2003;28: 19-22. | Meta-analysis of single dose oral tramadol plus acetaminophen in acute postoperative pain |
| 441 | Journal of Allergy and Clinical Immunology | 2003;112(1): 29-36. | Enhanced synergy between fluticasone propionate and salmeterol inhaled from a single inhaler versus separate inhalers |
| 442 | Contraception | 2003;68(6): 439-446. | Combined estimates of effectiveness of mifepristone 10 mg in emergency contraception. |
| 443 | Journal of the American College of Cardiology | 2003;41(9): 1529-1538. | Efficacy of angiotensin-converting enzyme inhibitors and beta-blockers in the management of left ventricular systolic dysfunction according to race, gender, and diabetic status: a meta-analysis of major clinical trials |
| 444 | Cochrane Database of Systematic Reviews | 2003;(3): CD003150. | Aroma therapy for dementia. |
| 445 | Cochrane Database of Systematic Reviews | 2003;(1): CD001904. | Carbamazepine versus phenobarbitone monotherapy for epilepsy |
| 446 | The Journal of the American Medical Association | 2002;288(16): 2015-2022. | Homocysteine and risk of ischemic heart disease and stroke: a meta-analysis |
| 447 | Journal of Internal Medicine | 2010;268(4):367-82 | Combined analyses and extended follow-up of two randomized controlled homocysteine-lowering B-vitamin trials |
| 448 | Carcinogenesis | 2002;23(8): 1343-1350. | Meta- and pooled analyses of the effects of glutathione S-transferase M1 polymorphisms and smoking on lung cancer risk. |
| 449 | Cancer Causes Control | 2002;13(8): 765-775. | A pooled analysis of case-control studies of thyroid cancer. VII. Cruciferous and other vegetables (International) |
| 450 | Annals of Internal Medicine | 2002;136(6): 438-448 | J-shaped relationship between blood pressure and mortality in hypertensive patients: new insights from a meta-analysis of individual-patient data |
| 451 | Gut | 2002;51(6): 864-869. | Retreatment with interferon plus ribavirin of chronic hepatitis C non-responders to interferon monotherapy: a meta-analysis of individual patient data |
| 452 | British Medical Journal | 2002;324(7329): 71-86. | Collaborative meta-analysis of randomised trials of antiplatelet therapy for prevention of death, myocardial infarction, and stroke in high risk patients. |
| 453 | Stroke | 2002;33(12): 2850-2857. | Oral Citicoline in Acute Ischemic Stroke: An Individual Patient Data Pooling Analysis of Clinical Trials. |
| 454 | Journal of Pain and Symptom Management | 2002;23(2): 121-130 | Combination analgesic efficacy: individual patient data meta-analysis of single-dose oral tramadol plus acetaminophen in acute postoperative pain |
| 455 | American journal of epidemiology | 2002;156(2): 95-109. | Pooled analysis and meta-analysis of glutathione S-transferase M1 and bladder cancer: a HuGE review. |
| 456 | Journal of Human Hypertension | 2002;20(1): 145-151. | Pulsatile blood pressure component as predictor of mortality in hypertension: a meta-analysis of clinical trial control groups |
| 457 | Hernia | 2002;6(3): 130-136. | Open mesh versus non-mesh repair of groin hernia: meta-analysis of randomised trials based on individual patient data. |
| 458 | Hernia | 2002;6(1): 2-10. | Laparoscopic versus open groin hernia repair: meta-analysis of randomised trials based on individual patient data. |
| 459 | British Journal of Cancer | 2002;87(11): 1234-1245. | Alcohol, tobacco and breast cancer--collaborative reanalysis of individual data from 53 epidemiological studies, including 58,515 women with breast cancer and 95,067 women without the disease |
| 460 | Chest | 2002121(6): 1916-1920. | Multilevel Likelihood Ratios for Identifying Exudative Pleural Effusions |
| 461 | Neurosurgery | 2002;51(5): 1101-1107. | The Probability of Sudden Death from Rupture of Intracranial Aneurysms: A Meta-analysis." |
| 462 | American Journal of Clinical Nutrition | 2000;72:1516-22 | Therapeutic effects of oral zinc in actue and persistent diarrhea in children in developing countries: pooled analysis of randomized controlled trials |
| 463 | Psychopharmacology (Berl) | 2002;164(3): 301-308. | Cluster analysis of symptoms during antidepressant treatment with Hypericum extract in mildly to moderately depressed out-patients. A meta-analysis of data from three randomized, placebo-controlled trials. |
| 464 | the Journals of gerontology | 2002;57(9): M599-604. | Exercise and lumbar spine bone mineral density in postmenopausal women: a meta-analysis of individual patient data. |
| 465 | Journal of the National Cancer Institute | 2002;94(8): 606-616. | Endogenous sex hormones and breast cancer in postmenopausal women: reanalysis of nine prospective studies |
| 466 | Leukemia | 2000;14(12):2257-66 | Long-term results of three randomized trials (58831, 58832, 58881) in childhood acute lymphoblastic leukemia: a CLCG-EORTC report. Children Leukemia Cooperative Group |
| 467 | American Heart Journal | 2002;143(2): 301-307. | Bisoprolol for the treatment of chronic heart failure: a meta-analysis on individual data of two placebo-controlled studies--CIBIS and CIBIS II. Cardiac Insufficiency Bisoprolol Study |
| 468 | The Lancet | 2002;360(9349): 1903-1913. | Age-specific relevance of usual blood pressure to vascular mortality: a meta-analysis of individual data for one million adults in 61 prospective studies. |
| 469 | British Journal of Clinical Pharmacology | 2002;54(3): 320-326. | Dose-response relationships between individual nonaspirin nonsteroidal anti-inflammatory drugs (NANSAIDs) and serious upper gastrointestinal bleeding: a meta-analysis based on individual patient data. |
| 470 | Journal of the National Cancer Institute | 2002;94(2): 116-128. | Pooled analysis of prognostic impact of urokinase-type plasminogen activator and its inhibitor PAI-1 in 8377 breast cancer patients |
| 471 | Epilepsia | 2002;43(5): 505-513. | Carbamazepine versus Valproate Monotherapy for Epilepsy: A Meta-analysis. |
| 472 | European Respiratory Journal | 2002;20(4): 846-852. | Efficacy of acupuncture in asthma: systematic review and meta-analysis of published data from 11 randomised controlled trials. |
| 473 | Journal of Hepatology | 2002;36(4): 480-487. | Corticosteroids improve short-term survival in patients with severe alcoholic hepatitis (AH): individual data analysis of the last three randomized placebo controlled double blind trials of corticosteroids in severe AH." |
| 474 | Annals of Internal Medicine | 2002;136(1): 13-24. | Risk factors for Helicobacter pylori resistance in the United States: the surveillance of H. pylori antimicrobial resistance partnership (SHARP) study, 1993-1999. |
| 475 | Cochrane Database of Systematic Reviews | 2002;(4): CD002197. | Open mesh versus non-mesh for groin hernia repair. |
| 476 | The Lancet | 2002;359(9311): 1011-1018. | Chemotherapy in adult high-grade glioma: a systematic review and meta-analysis of individual patient data from 12 randomised trials |
| 477 | Annals of Surgery | 2002;235(3): 322-332. | Repair of Groin Hernia With Synthetic Mesh: Meta-Analysis of Randomized Controlled Trials. |
| 478 | Cochrane Database of Systematic Reviews | 2002;(2): CD001911. | Carbamazepine versus phenytoin monotherapy for epilepsy. |
| 479 | The Journal of the American Medical Association | 2002;288(19): 2441-2448. | Oral Anticoagulants vs Aspirin in Nonvalvular Atrial Fibrillation: An Individual Patient Meta-analysis |
| 480 | Biology of the Neonate | 2002;81(3): 182-187. | Prophylactic administration of porcine-derived lung surfactant is a significant factor in reducing the odds for peri-intraventricular haemorrhage in premature infants |
| 481 | Journal of Clinical Oncology | 2002;20(22): 4448-4452. | Prognostic factors for relapse in stage I seminoma managed by surveillance: a pooled analysis |
| 482 | International Journal of Geriatric Psychiatry | 2002;17(2): 175-183. | The effect of selegiline in the treatment of people with Alzheimer's disease: a meta-analysis of published trials |
| 483 | Transactions of the Royal Society of Tropical Medicine and Hygiene | 2001;95(6): 637-650. | A meta-analysis using individual patient data of trials comparing artemether with quinine in the treatment of severe falciparum malaria. |
| 484 | The Lancet | 2001;358(9290): 1291-1304. | Adjuvant radiotherapy for rectal cancer: a systematic overview of 8,507 patients from 22 randomised trials. |
| 485 | AIDS | 2001;15(3): 357-368. | Duration of ruptured membranes and vertical transmission of HIV-1: a meta-analysis from 15 prospective cohort studies |
| 486 | Kidney Int | 2001;59(1): 260-269 | Effect of lipid reduction on the progression of renal disease: a meta-analysis. |
| 487 | International Journal of Hygiene and Environmental Health | 2001;204(1): 39-42. | International collaborative study on genetic susceptibility to environmental carcinogens (GSEC): an update |
| 488 | Annals of Internal Medicine | 2001;135(9): 782-795. | Effects of CCR5-Delta32, CCR2-64I, and SDF-1 3'A alleles on HIV-1 disease progression: An international meta-analysis of individual-patient data |
| 489 | Annals of Internal Medicine | 2001;135(2): 73-87. | Angiotensin-Converting Enzyme Inhibitors and Progression of Nondiabetic Renal Disease: A Meta-Analysis of Patient-Level Data. |
| 490 | Journal of Clinical Oncology | 2001;19(2): 343-353. | Combined tamoxifen and luteinizing hormone-releasing hormone (LHRH) agonist versus LHRH agonist alone in premenopausal advanced breast cancer: a meta-analysis of four randomized trials |
| 491 | Thrombosis Research | 2001;102(4):295-309. | Low molecular weight heparin and unfractionated heparin in thrombosis prophylaxis: meta-analysis based on original patient data. |
| 492 | British Journal of Cancer | 2001;84(3): 303-307. | Survival in patients with intermediate or high grade non-Hodgkin's lymphoma: meta-analysis of randomized studies comparing third generation regimens with CHOP |
| 493 | British Medical Journal | 2001;323(7304): 75-81 | A score for predicting risk of death from cardiovascular disease in adults with raised blood pressure, based on individual patient data from randomised controlled trials |
| 494 | Oncology Reports | 2001;8(3): 697-703. | An individual patient data meta-analysis of long supported adjuvant chemotherapy with oral carmofur in patients with curatively resected colorectal cancer http://www.spandidos-publications.com/or/8/3/697 |
| 495 | New England Journal of Medicine | 2001;345(15): 1091-1097 | A pooled analysis of adjuvant chemotherapy for resected colon cancer in elderly patients. |
| 496 | Cochrane Database of Systematic Reviews | 2001;(4): CD002217. | Phenobarbitone versus phenytoin monotherapy for partial onset seizures and generalized onset tonic-clonic seizures |
| 497 | Annals of Internal Medicine | 2001;134(5): 370-379. | Should All Patients with Type 1 Diabetes Mellitus and Microalbuminuria Receive Angiotensin-Converting Enzyme Inhibitors?: A Meta-Analysis of Individual Patient Data |
| 498 | British Journal of Haematology | 2001;113(4-II): 1020-1034. | Interferon as therapy for multiple myeloma: an individual patient data overview of 24 randomized trials and 4012 patients. |
| 499 | British Journal of Cancer | 2001;84(5): 611-620. | Alpha-interferon does not increase the efficacy of 5-fluorouracil in advanced colorectal cancer |
| 500 | Psychopharmacology (Berl) | 2005;178(2-3):167-73 | Predictors of acamprosate efficacy: results from a pooled analysis of seven European trials including 1485 alcohol-dependent patients |
| 501 | British Medical Journal | 1998;316(7135):894-8 | Lowering blood homocysteine with folic acid based supplements: meta-analysis of randomised trials. Homocysteine Lowering Trialists' Collaboration |
| 502 | Cochrane Database of Systematic Reviews | 2000;(4): CD002805. | Cranial irradiation for preventing brain metastases of small cell lung cancer in patients in complete remission |
| 503 | British Journal of Haematology | 2000;110(3): 573-576. | Hydroxyurea versus busulphan for chronic myeloid leukaemia: an individual patient data meta-analysis of three randomized trials. Chronic myeloid leukemia trialists' collaborative group |
| 504 | The Lancet | 2000;355(9217): 1757-1770. | Favourable and unfavourable effects on long-term survival of radiotherapy for early breast cancer: an overview of the randomised trials. Early Breast Cancer Trialists' Collaborative Group |
| 505 | Cochrane Database of Systematic Reviews | 2000;(2): CD001419. | Adjuvant chemotherapy for localised resectable soft tissue sarcoma in adults. Sarcoma Meta-analysis Collaboration (SMAC). |
| 506 | Cochrane Database of Systematic Reviews | 2000;(2): CD002139. | Chemotherapy for non-small cell lung cancer. |
| 507 | Cochrane Database of Systematic Reviews | 2000;(2): CD001545. | Palliative chemotherapy for advanced or metastatic colorectal cancer. Colorectal Meta-analysis Collaboration |
| 508 | Cochrane Database of Systematic Reviews | 1999;(2): CD001426. | Neoadjuvant cisplatin for advanced bladder cancer. |
| 509 | AIDS Research and Human Retroviruses | 2000;16(12): 1123-1133. | Human immunodeficiency virus type 1 RNA level and CD4 count as prognostic markers and surrogate end points: a meta-analysis. HIV Surrogate Marker Collaborative Group |
| 510 | Cochrane Database of Systematic Reviews | 2000;(2): CD001941. | Nocturnal mechanical ventilation for chronic hypoventilation in patients with neuromuscular and chest wall disorders. |
| 511 | Cochrane Database of Systematic Reviews | 2000;(2): CD001799. | Preoperative radiotherapy for esophageal carcinoma. Oeosphageal Cancer Collaborative Group |
| 512 | Archives of Internal Medicine | 2000;160(8): 1085-1089. | Pulse pressure not mean pressure determines cardiovascular risk in older hypertensive patients |
| 513 | The Lancet | 2000;356(9227): 373-378. | Relation between tumour response to first-line chemotherapy and survival in advanced colorectal cancer: a meta-analysis. Meta-Analysis Group in Cancer |
| 514 | British Medical Journal | 2000;321(7260): 531-535. | Palliative chemotherapy for advanced colorectal cancer: systematic review and meta-analysis. |
| 515 | The Journal of the American Medical Association | 1998;279(7):535-40 | Alcohol and breast cancer in women: a pooled analysis of cohort studies |
| 516 | Stroke | 2000;31(7): 1555-1560. | Streptokinase in Acute Ischemic Stroke: An Individual Patient Data Meta-Analysis: The Thrombolysis in Acute Stroke Pooling Project. |
| 517 | Cochrane Database of Systematic Reviews | 2000;(3): CD002039. | Immediate versus deferred zidovudine (AZT) in asymptomatic or mildly symptomatic HIV infected adults. |
| 518 | Cochrane Database of Systematic Reviews | 2000;(3): CD002038. | Zidovudine (AZT) versus AZT plus didanosine (ddI) versus AZT plus zalcitabine (ddC) in HIV infected adults |
| 519 | The Lancet | 2000;355(9215): 1575-1581. | Long-term ACE-inhibitor therapy in patients with heart failure or left-ventricular dysfunction: a systematic overview of data from individual patients. ACE-Inhibitor Myocardial Infarction Collaborative Group |
| 520 | Epidemiology | 2000;11(6): 624-634. | A Pooled Analysis of Magnetic Fields, Wire Codes, and Childhood Leukemia |
| 521 | Chest | 2000;117(1): 87-95. | Pleural Fluid pH as a Predictor of Pleurodesis Failure*: Analysis of Primary Data. |
| 522 | Intensive Care Medicine | 2000;26 Suppl 1: S38-44. | Antibiotic prophylaxis in intensive care units: meta-analyses versus clinical practice |
| 523 | Archives of Dermatology | 2000;136(12): 1531-1535. | Risk factors for delayed healing of neuropathic diabetic foot ulcers: a pooled analysis |
| 524 | Biomedical and Environmental Sciences | 2002;15(3):245-52 | Effect of body mass index on all-cause mortality and incidence of cardiovascular diseases--report for meta-analysis of prospective studies open optimal cut-off points of body mass index in Chinese adults |
| 525 | The Lancet | 2000;356(9246): 1955-1964. | Effects of ACE inhibitors, calcium antagonists, and other blood-pressure-lowering drugs: results of prospectively designed overviews of randomised trials. |
| 526 | European Journal of Contraception and Reproductive Health Care | 2000;5(4): 265-274. | The effects of age, body mass index, smoking and general health on the risk of venous thromboembolism in users of combined oral contraceptives |
| 527 | The Lancet | 2000;355(9208): 949-955. | Chemotherapy added to locoregional treatment for head and neck squamous-cell carcinoma: three meta-analyses of updated individual data. MACH-NC Collaborative Group. Meta-Analysis of Chemotherapy on Head and Neck Cancer. |
| 528 | Hepatology | 2000;32(5): 1131-1137. | Impact of Interferon Alfa-2b and Ribavirin on Progression of Liver Fibrosis in Patients With Chronic Hepatitis C. |
| 529 | Clinical Therapeutics | 2000;22(12): 1562-1574. | Cost-efficacy comparison of inhaled fluticasone propionate and budesonide in the treatment of asthma |
| 530 | The Lancet | 1999;353(9169): 2014-2025. | Zidovudine, didanosine, and zalcitabine in the treatment of HIV infection: meta-analyses of the randomised evidence. HIV Trialists' Collaborative Group |
| 531 | New England Journal of Medicine | 1999;340(13): 977-987. | The Mode of Delivery and the Risk of Vertical Transmission of Human Immunodeficiency Virus Type 1 - A Meta-Analysis of 15 Prospective Cohort Studies. |
| 532 | Hepatology | 1999;30(3): 801-807. | Chronic hepatitis C: interferon retreatment of relapsers. A meta-analysis of individual patient data. European Concerted Action on Viral Hepatitis (EUROHEP). |
| 533 | Annals of Oncology | 1999;10(9): 1035-1041. | Systematic review and meta-analysis of corticosteroids for the resolution of malignant bowel obstruction in advanced gynaecological and gastrointestinal cancers. Systematic Review Steering Committee |
| 534 | Schizophrenia Bulletin | 1999;25(3): 413-423. | Schizophrenia and complications of pregnancy and labor: an individual patient data meta-analysis. |
| 535 | Circulation | 1999;100(18): e88-94. | Identification of risk factors in hypertensive patients: contribution of randomized controlled trials through an individual patient database |
| 536 | Graefe's Archive for Clinical and Experimental Ophthalmology | 1999;237(9): 705-713. | Meta-analysis of six clinical phase III studies comparing lomefloxacin 0.3% eye drops twice daily to five standard antibiotics in patients with acute bacterial conjunctivitis. |
| 537 | Resuscitation | 1999;41(3): 249-256. | Effect of active compression-decompression resuscitation (ACD-CPR) on survival: a combined analysis using individual patient data |
| 538 | Cancer Causes Control | 1999;10(2): 143-155. | A pooled analysis of case-control studies of thyroid cancer. II. Menstrual and reproductive factors |
| 539 | Journal of Acquired Immune Deficiency Syndromes | 1999;22(3): 260-266. | Meta-analysis of two randomized controlled trials comparing combined zidovudine and didanosine therapy with combined zidovudine, didanosine, and nevirapine therapy in patients with HIV. |
| 540 | Japanese Journal of Clinical Oncology | 1999;29(2): 78-86 | Adjuvant therapy with oral fluoropyrimidines as main chemotherapeutic agents after curative resection for colorectal cancer: individual patient data meta-analysis of randomized trials |
| 541 | Annals of Oncology | 1999;10(11): 1317-1320. | Survival impact of chemotherapy in patients with colorectal metastases confined to the liver: a re-analysis of 1458 non-operable patients randomised in 22 trials and 4 meta-analyses. Meta-Analysis Group in Cancer |
| 542 | Journal of Clinical Oncology | 1998;16(12): 3832-3842. | Combination chemotherapy versus melphalan plus prednisone as treatment for multiple myeloma: an overview of 6,633 patients from 27 randomized trials. |
| 543 | Journal of Clinical Oncology | 1998;16(11): 3537-3541. | Toxicity of fluorouracil in patients with advanced colorectal cancer: effect of administration schedule and prognostic factors. Meta-Analysis Group In Cancer http://jco.ascopubs.org/content/16/11/3537.long |
| 544 | The Lancet | 1998;352(9132): 930-942. | Polychemotherapy for early breast cancer: an overview of the randomised trials. Early Breast Cancer Trialists' Collaborative Group. |
| 545 | Circulation | 1998;97(22): 2202-2212. | Indications for ACE inhibitors in the early treatment of acute myocardial infarction: systematic overview of individual data from 100,000 patients in randomized trials. ACE Inhibitor Myocardial Infarction Collaborative Group |
| 546 | The Lancet | 1998;351(9114): 1451-1467. | Tamoxifen for early breast cancer: an overview of the randomised trials. Early Breast Cancer Trialists' Collaborative Group. |
| 547 | Journal of Clinical Oncology | 1998;16(1): 301-308. | Efficacy of intravenous continuous infusion of fluorouracil compared with bolus administration in advanced colorectal cancer. |
| 548 | The Lancet | 1998;352(9124): 257-263. | Postoperative radiotherapy in non-small-cell lung cancer: systematic review and meta-analysis of individual patient data from nine randomised controlled trials. |
| 549 | British Journal of Cancer | 1998;78(11): 1479-1487. | Chemotherapy in advanced ovarian cancer: four systematic meta-analyses of individual patient data from 37 randomized trials. Advanced Ovarian Cancer Trialists' Group |
| 550 | International Journal of Radiation Oncology*Biology*Physics | 1998;41(3): 579-583. | Preoperative radiotherapy in esophageal carcinoma: a meta-analysis using individual patient data (Oesophageal Cancer Collaborative Group). |
| 551 | British Medical Journal | 1998;316(7140): 1275-1285. | Effectiveness of antibiotic prophylaxis in critically ill adult patients: systematic review of randomised controlled trials |
| 552 | Advances in Therapy | 1998;(4): 241-251. | Tizanidine treatment of spasticity: a meta-analysis of controlled, double-blind, comparative studies with baclofen and diazepam. |
| 553 | Journal of the National Cancer Institute | 1998;90(11): 850-858 | A new prognostic score for survival of patients with chronic myeloid leukemia treated with interferon alfa. Writing Committee for the Collaborative CML Prognostic Factors Project Group. |
| 554 | AIDS | 1998;12(14): 1823-1832. | CD4 cell count as a surrogate endpoint in HIV clinical trials: a meta-analysis of studies of the AIDS Clinical Trials Group. |
| 555 | Journal of Infectious Diseases | 1998;178(2): 349-359. | Clinical Efficacy of High-Dose Acyclovir in Patients with Human Immunodeficiency Virus Infection: A Meta-Analysis of Randomized Individual Patient Data |
| 556 | Public Health Nutrition | 1998;1(1): 33-41. | Mortality in vegetarians and non-vegetarians: a collaborative analysis of 8300 deaths among 76,000 men and women in five prospective studies |
| 557 | The Lancet | 1998;352(9128): 597-600. | International multicentre pooled analysis of late postnatal mother-to-child transmission of HIV-1 infection. Ghent International Working Group on Mother-to-Child Transmission of HIV |
| 558 | Journal of Clinical Oncology | 1998;16(3): 818-829. | Meta-analysis of chemotherapy versus combined modality treatment trials in Hodgkin's disease. |
| 559 | Journal of General Internal Medicine | 1998;13(9): 600-606. | Quinine for Nocturnal Leg Cramps: A Meta-Analysis Including Unpublished Data. |
| 560 | The Journal of the American Medical Association | 1998;280(20): 1777-1782. | Cholinesterase inhibition for Alzheimer disease: a meta-analysis of the tacrine trials. Dementia Trialists' Collaboration. |
| 561 | Journal of Clinical Oncology | 1998;16(3): 830-843. | Influence of more extensive radiotherapy and adjuvant chemotherapy on long-term outcome of early-stage Hodgkin's disease: a meta-analysis of 23 randomized trials involving 3,888 patients. |
| 562 | Annals of Internal Medicine | 1998;128(10): 817-826. | The Effect of Antilymphocyte Induction Therapy on Renal Allograft Survival: A Meta-Analysis of Individual Patient-Level Data. |
| 563 | Obstetrical & Gynecological Survey | 1998;53(3): 133-134. | Obstetric Complications and Age at Onset in Schizophrenia: An International Collaborative Meta-Analysis of Individual Patient Data. |
| 564 | The Lancet | 1997;350(9084): 1047-1059. | Breast cancer and hormone replacement therapy: collaborative reanalysis of data from 51 epidemiological studies of 52,705 women with breast cancer and 108,411 women without breast cancer. |
| 565 | The Lancet | 1997;350(9092): 1647-1654. | Adjuvant chemotherapy for localised resectable soft-tissue sarcoma of adults: meta-analysis of individual data. |
| 566 | The Lancet | 1997;350(9089): 1417-1424. | Effect of prophylactic amiodarone on mortality after acute myocardial infarction and in congestive heart failure: meta-analysis of individual data from 6500 patients in randomised trials. |
| 567 | Stroke | 1997;28(11): 2139-2144. | How do stroke units improve patient outcomes? A collaborative systematic review of the randomized trials. Stroke Unit Trialists Collaboration |
| 568 | Journal of the National Cancer Institute | 1997;89(21): 1616-1620. | Interferon alfa versus chemotherapy for chronic myeloid leukemia: a meta-analysis of seven randomized trials: Chronic Myeloid Leukemia Trialists' Collaborative Group |
| 569 | Archives of Internal Medicine | 1997;157(11): 1237-1240. | The efficacy of aspirin in patients with atrial fibrillation. Analysis of pooled data from 3 randomized trials. The Atrial Fibrillation Investigators |
| 570 | Journal of Clinical Oncology | 1997;15(7): 2526-2535. | Overview of randomized perioperative polychemotherapy trials in women with early-stage breast cancer |
| 571 | Breast Cancer Research & Treatment | 1997;44(3): 201-210 | An individual patient-based meta-analysis of tamoxifen versus ovarian ablation as first line endocrine therapy for premenopausal women with metastatic breast cancer |
| 572 | Pediatrics | 1997;100(1): E4. | Mortality, severe respiratory distress syndrome, and chronic lung disease of the newborn are reduced more after prophylactic than after therapeutic administration of the surfactant Curosurf |
| 573 | Stroke | 1997;28(12): 2557-2562. | Effect of antihypertensive treatment in patients having already suffered from stroke. Gathering the evidence. The INDANA (INdividual Data ANalysis of Antihypertensive intervention trials) Project Collaborators |
| 574 | Annals of Internal Medicine | 1997;126(10): 761-767 | Effect of antihypertensive drug treatment on cardiovascular outcomes in women and men. A meta-analysis of individual patient data from randomized, controlled trials. The INDANA Investigators |
| 575 | The Journal of the American Medical Association | 1997;277(4): 325-332. | Effects of acadesine on myocardial infarction, stroke, and death following surgery. A meta-analysis of the 5 international randomized trials. The Multicenter Study of Perioperative Ischemia (McSPI) Research Group. |
| 576 | Pain | 1997;69(3): 287-294. | Single-patient data meta-analysis of 3453 postoperative patients: oral tramadol versus placebo, codeine and combination analgesics |
| 577 | The cancer journal from Scientific American | 1997;3(1): 6-12. | Breast-conserving therapy vs mastectomy in early-stage breast cancer: a meta-analysis of 10-year survival. |
| 578 | Antiviral Therapy | 1997;2(4): 237-247. | Meta-analysis of five randomized controlled trials comparing continuation of zidovudine versus switching to didanosine in HIV-infected individuals |
| 579 | Scandinavian Journal of Gastroenterology - Supplement | 1997;223: 46-49. | Antiviral therapy of hepatitis C. |
| 580 | Journal of Hepatology | 1997;26(5): 961-966. | Ribavirin enhances the efficacy but not the adverse effects of interferon in chronic hepatitis C. Meta-analysis of individual patient data from European centers |
| 581 | British Medical Journal | 1995;310:13-7 | Meta-Analysis of Efficacy of Quinine for Treatment of Nocturnal Leg Cramps in Elderly People. |
| 582 | Acta Anaesthesiol Scand Suppl | 1997;110: 77-79. | Efficacy of hypertonic saline dextran (HSD) in patients with traumatic hypotension: meta-analysis of individual patient data. |
| 583 | The Lancet | 1996;348(9036): 1189-1196. | Ovarian ablation in early breast cancer: overview of the randomised trials. Early Breast Cancer Trialists' Collaborative Group |
| 584 | The Lancet | 1996;347(9017): 1713-1727. | Breast cancer and hormonal contraceptives: collaborative reanalysis of individual data on 53 297 women with breast cancer and 100 239 women without breast cancer from 54 epidemiological studies. Collaborative Group on Hormonal Factors in Breast Cancer |
| 585 | Journal of the National Cancer Institute | 1996;88(5): 252-258. | Reappraisal of hepatic arterial infusion in the treatment of nonresectable liver metastases from colorectal cancer. |
| 586 | Journal of Parenteral and Enteral Nutrition (JPEN) | 1996;20(2): 159-164. | Overview of randomized clinical trials of oral branched-chain amino acid treatment in chronic hepatic encephalopathy |
| 587 | Journal of Hepatology | 1996;25(6): 795-802. | Relation between treatment efficacy and cumulative dose of alpha interferon in chronic hepatitis B. European Concerted Action on Viral Hepatitis (Eurohep). |
| 588 | Diabetes Care | 1996;19(10): 1091-1096. | The efficacy of tolrestat in the treatment of diabetic peripheral neuropathy. A meta-analysis of individual patient data. |
| 589 | Journal of Urology | 1996; 156(6): 1934-1940. | A combined analysis of European Organization for Research and Treatment of Cancer, and Medical Research Council randomized clinical trials for the prophylactic treatment of stage TaT1 bladder cancer. |
| 590 | The Journal of the American Medical Association | 1996;276(15): 1246-1252. | A clinical approach for the diagnosis of diabetes mellitus: an analysis using glycosylated hemoglobin levels. Meta-analysis Research Group on the Diagnosis of Diabetes Using Glycated Hemoglobin Levels. |
| 591 | Antimicrobial Agents and Chemotherapy | 1996;40(3): 575-580. | Factors influencing elimination and distribution of fleroxacin: metaanalysis of individual data from 10 pharmacokinetic studies |
| 592 | Cancer | 1996;77(11): 2206-2212. | Prognostic value of preoperative immunosuppressive acidic protein in patients with gastric carcinoma. Findings from three independent clinical trials. Tumor Marker Committee for the Study Group of Immunochemotherapy with PSK for Gastric Cancer |
| 593 | New England Journal of Medicine | 1995;333(22): 1444-1455. | Effects of radiotherapy and surgery in early breast cancer. An overview of the randomized trials. Early Breast Cancer Trialists' Collaborative Group. |
| 594 | The Lancet | 1995;346(8970): 265-269. | Maximum androgen blockade in advanced prostate cancer: an overview of 22 randomised trials with 3283 deaths in 5710 patients. Prostate Cancer Trialists' Collaborative Group |
| 595 | British Journal of Urology | 1995;75(2): 206-213. | Does neoadjuvant cisplatin-based chemotherapy improve the survival of patients with locally advanced bladder cancer: a meta-analysis of individual patient data from randomized clinical trials. |
| 596 | Journal of Clinical Gastroenterology | 1995;21(2): 134-138. | Perioperative blood transfusion does not prevent recurrence in Crohn's disease. A pooled analysis |
| 597 | Journal of Clinical Oncology | 1994;12(5): 960-969. | Meta-analysis of randomized trials testing the biochemical modulation of fluorouracil by methotrexate in metastatic colorectal cancer. |
| 598 | Science of the Total Environment | 1994;152(2): 169-177 | Estimation of pooled reference values for cadmium in blood using meta-analysis and TRACY criteria. |
| 599 | Anticancer Research | 1994;14(1B): 333-335. | Effect of thoracic radiotherapy on mortality in limited small cell lung cancer. A meta-analysis of 13 randomized trials among 2,140 patients. |
| 600 | American Journal of Reproductive Immunology | 1994;32(4): 294-302 | The effectiveness of allogeneic leukocyte immunization in unexplained primary recurrent spontaneous abortion. Recurrent Miscarriage Immunotherapy Trialists Group. |
| 601 | Addiction | 1994;89(9): 1143-1156. | Relationships of measures of alcohol consumption with alcohol-related problems in multiple studies: a research synthesis from the collaborative alcohol-related longitudinal project |
| 602 | Journal of Hepatology | 1994;21(4): 646-655. | The treatment effect of alpha interferon in chronic hepatitis B is independent of pre-treatment variables. Results based on individual patient data from 10 clinical controlled trials. European Concerted Action on Viral Hepatitis (Eurohep). |
| 603 | Forensic Science International | 1994;66(3): 159-174. | Human postmortem interval estimation from vitreous potassium: an analysis of original data from six different studies. |
| 604 | Journal of Pediatrics | 1994;124(4): 574-584. | Risk factors for seizure recurrence in children with febrile seizures: a pooled analysis of individual patient data from five studies. |
| 605 | The Annals of Thoracic Surgery | 1994;58(2): 585-593. | Traumatic aortic rupture: twenty-year metaanalysis of mortality and risk of paraplegia. |
| 606 | Online Journal of Current Clinical Trials | 1994;145: [3987 words; 3938 paragraphs]. | Ten-year results of the randomized control trials of coronary artery bypass graft surgery: tabular data compiled by the collaborative effort of the original trial investigators. Part 1 of 2 |
| 607 | Arthritis & Rheumatism | 1993;36(6): 804-810. | Differential effects of diclofenac and aspirin on serum glutamic oxaloacetic transaminase elevations in patients with rheumatoid arthritis and osteoarthritis |
| 608 | Bull Cancer | 1993;80(2): 146-151. | The results of a quantitative overview of chemotherapy in advanced ovarian cancer: what can we learn? |
| 609 | Journal of the National Cancer Institute | 1992;84(6): 430-435. | Serum alpha-tocopherol concentration in relation to subsequent colorectal cancer: pooled data from five cohorts |
| 610 | New England Journal of Medicine | 1992;327(23): 1618-1624. | A meta-analysis of thoracic radiotherapy for small-cell lung cancer. |
| 611 | American journal of epidemiology | 1992;136(10): 1212-1220. | Characteristics relating to ovarian cancer risk: collaborative analysis of 12 US case-control studies. IV. The pathogenesis of epithelial ovarian cancer. Collaborative Ovarian Cancer Group http://aje.oxfordjournals.org/content/136/10/1212.full.pdf+html |
| 612 | New England Journal of Medicine | 1999;341:476-484 | Prophylatic cranial irradiation for patients with small-cell lung cancer in complete remision |
| 613 | Journal of Clinical Oncology | 1992;10:896-34 | Modulation of fluorouracil by leucovorin in patients with advanced colorectal cancer: evidence in terms of response rate |
| 614 | The Lancet | 1996;347:1783-8 | Duration and intensity of maintenance chemotherapy in acute lymphoblastic leukemia: overview of 42 trials involving 12000 randomised children |
| 615 | Transactions of the American Ophthalmological Society | 1998;96:431-50 | The therapy of amblyopia: an analysis of the results of amblyopia therapy utilizing the pooled data of published studies |
| 616 | Health Technology Assessment | 2001;5:1-75 | Systematic reviews of the effectiveness of day care for people with severe mental disorders |
| 617 | Health Technology Assessment | 2005;9(14):1-203, iii-iv. | Laparoscopic surgery for inguinal hernia repair: systematic review of effectiveness and economic evaluation. |
| 618 | British Medical Journal | 1991;303:884-893 | Chemotherapy in advanced ovarian cancer: an overview of randoised clinical trials |
| 619 | American journal of epidemiology | 1992;136:1184-1203 | Characteristics relating to ovarian cancer risk: collaborative analysis of 12 US case-control studies: II invasive epithelial ovarian cancers in white women |
| 620 | Obstetric review | 2011;12(9):680-7. | Body mass index, waist circumference and waist-hip ratio: which is the better discriminator of cardiovascular disease mortality risk?: evidence from an individual-participant meta-analysis of 82 864 participants from nine cohort studies. |
| 621 | The Lancet | 2009;373(9674): 1532–1542. | Recombinant human erythropoiesis-stimulating agents and mortality in patients with cancer. |
| 622 | Aging Clinical and Experimental Research | 2005;17(2):150-6 | Vertebral fracture risk reduction with risedronate in post-menopausal women with osteoprosis |
| 623 | Journal of Clinical Oncology | 2004;22(10):1797-806 | Pooled analysis of fluorouracilbased adjuvant therapy for stage II and III colon cancer |
| 624 | Osteoporosis International | 2005;16(5):475-82. | Risedronate decreases fracture risk in patients selected solely on the basis of prior vertebral fracture |
| 625 | The Lancet | 2001;358(9294):1668-75 | Oral triptans (serotonin 5-HT(1B/1D) agonists) in acute migraine treatment |
| 626 | The Lancet | 1993;341(8851):973-8 | breast cancer screening with mammography: overview of swedish randomized trials |
| 627 | The Lancet | 1992;339. No 8784 | Systemic treatment of early breast cancer by hormonal, cytotoxic, or immune therapy: 133 randomised trials involving 31000 recurrences and 24000 deaths among 75000 women |
| 628 | The Journal of the American Medical Association | 2012;307(4):382-390 | Association Between BRCA1 and BRCA2 Mutations and Survival in Women With Invasive Epithelial Ovarian Cancer |
| 629 | Biological Psychiatry | 2002;52(12):1166-74 | Efficacy efficacy between venlafaxine and SSRIs: a pooled analysis of patients with depression |
| 630 | British Medical Journal | 2011;342:d3036 | Influence of preceding length of anticoagulant treatment and initial presentation of venous thromboembolism on risk of recurrence after stopping treatment: analysis of individual participants' data from seven trials |
| 631 | European Heart Journal | 2006;27:779-788 | Does time matter? A pooled analysis of randomized clinical trials comparing primary percutaneous coronary intervention and in-hospital fibrinolysis in acute myocardial infarction patients |
| 632 | Journal of the American Society of Nephrology | 2004;15:1307-1315. | Chronic kidney disease as a risk factor for cardiovascular disease and all-cause mortality: a pooled analysis of community-based studies |
| 633 | Current Opinion in Oncology | 2007;19(3):188-194 | Individual patients' data meta-analyses in head and neck cancer |
| 634 | European journal of cancer | 2006;42:827-834 | Efficacy and tolerability of chemotherapy in elderly patients with advanced oesophage-gastric cancer: a pooled analysis of three clincial trials |
| 635 | Annals of Epidemiology | 2005;15(2):87-97 | Body mass index and mortality: a meta-analysis based on person-level data from twenty-six observational studies |
| 636 | American Journal of Cardiology | 2007;99(1):91-98. | Risk of cardiovascular events in patients receiving celecoxib: a meta-analysis of randomized clinical trials. |
| 637 | Clinical Therapeutics | 2000;22(9):1035-48 | Effect of early intervention with sumatriptan on migraine pain: retrospective analyses of data from three clincial trials |
| 638 | European Radiology | 1995;5,S79-S84 | Renal tolerance of iotrolan 280-a meta-analysis of 14 double-blind studies |
| 639 | The Lancet | 2005;365(9458):501-6 | Early supported discharge services for stroke patients: a meta-analysis of individual patients' data |
| 640 | The Lancet | 2003;361:1927-34 | Neoadjuvant chemotherapy in invasive bladder cancer: a systematic review and meta-analysis |
| 641 | The Lancet | 2002;360:603-9 | Infrared ear thermometry compared with rectal thermometry in children: a systematic review |
| 642 | Journal of Neurosurgery | 2003;99:666-73 | Patient age and outcome following severe traumatic brain injury: an analysis of 5600 paitents |
| 643 | Journal of Clinical Psychiatry | 1998;59(10):502-8 | Effects of venlafaxine on blood pressure: a meta-analysis of original data from 3744 depressed patients |
| 644 | Acute Cardiac Care | 2011;13(1):14-20 | Early revascularization is beneficial across all ages and a wide spectrum of cardiogenic shock severity: A pooled analysis of trials |
| 645 | AIDS | 2006;20(7):1039-1049 | Clinical safety of HIV lipopeptides used as vaccines in healthy volunteers and HIV-infected adults |
| 646 | AIDS and Behavior | 2012;16(1):79-85 | Mother's CD4+ count moderates the risk associated with higher parity for late postnatal HIV-free survival of breastfed children: an individual patient data meta-analysis of randomized controlled trials |
| 647 | Alcoholism: Clinical and Experimental research | 2012;36(3):497-508. | Acamprosate for alcohol dependence: a sex-specific meta-analysis based on individual patient data. |
| 648 | Alzheimer Disease & Associated Disorders | 2009;23(4):357-364. | Rates of cognitive change in Alzheimer disease: observations across a decade of placebo-controlled clinical trials with donepezil. |
| 649 | American Heart Journal | 2011;161(2):283-290 | The influence of time from symptom onset and reperfusion strategy on 1-year survival in ST-elevation myocardial infarction: a pooled analysis of an early fibrinolytic strategy versus primary percutaneous coronary intervention from CAPTIM and WEST |
| 650 | American Journal of Clinical Nutrition | 1999;69(6):1117-1122 | Meta-analysis of resting metabolic rate in formerly obese subjects |
| 651 | American Journal of Kidney Diseases | 2011;58(6):928-938 | Therapeutic response to vasoconstrictors in hepatorenal syndrome parallels increase in mean arterial pressure: a pooled analysis of clinical trials |
| 652 | Annals of Epidemiology | 2005;15(5):405-413 | A comparison of lipid variables as predictors of cardiovascular disease in the Asia Pacific region |
| 653 | American Journal of Kidney Diseases | 2004;44(2):198-206 | Kidney disease as a risk factor for recurrent cardiovascular disease and mortality |
| 654 | Annals of Oncology | 2007;18(10):1652-1659. | Increased survival using platinum analog combined with gemcitabine as compared to single-agent gemcitabine in advanced pancreatic cancer: pooled analysis of two randomized trials, the GERCOR/GISCAD intergroup study and a German multicenter study. |
| 655 | Archives of General Psychiatry | 2006;63(3):332-339 | Suicidality in pediatric patients treated with antidepressant drugs |
| 656 | Archives of Internal Medicine | 1994;154(13):1449-1457 | Risk factors for stroke and efficacy of antithrombotic therapy in atrial fibrillation: Analysis of pooled data from five randomized controlled trials |
| 657 | Archives of Internal Medicine | 2012;172(8):611-621. | Drug-eluting vs bare-metal stents in primary angioplasty: a pooled patient-level meta-analysis of randomized trials. |
| 658 | Archives of Internal Medicine | 2007;167(11):1130-1136 | Cardiovascular disease and subsequent kidney disease |
| 659 | Arthritis care & research | 2011;63(7):1055-1061 | Health-related quality of life in patients with newly diagnosed antineutrophil cytoplasmic antibody-associated vasculitis |
| 660 | Biological Psychiatry | 2011;69(4):326-335 | Structural magnetic resonance imaging in bipolar disorder: an international collaborative mega-analysis of individual adult patient data |
| 661 | American journal of epidemiology | 2010;172(7):752-61 | A pooled analysis of extremely low-frequency magnetic fields and childhood brain tumors |
| 662 | Annals of Medicine | 2009;41(2):128-38 | Joint analysis of individual participants' data from 17 studies on the association of the IL6 variant -174G>C with circulating glucose levels, interleukin-6 levels, and body mass index |
| 663 | BMC Public Health | 2009;9:294 | Impact of cigarette smoking on the relationship between body mass index and coronary heart disease: a pooled analysis of 3264 stroke and 2706 CHD events in 378579 individuals in the Asia Pacific region |
| 664 | Journal of Clinical Oncology | 2005;23(6):1118-24 | Long-term survival after cisplatin-based induction chemotherapy and radiotherapy for nasopharyngeal carcinoma: a pooled data analysis of two phase III trials |
| 665 | BMC Women's Health | 2004;4(5) | Rofecoxib for dysmenorrhoea: Meta-analysis using individual patient data |
| 666 | British Medical Journal | 1995;311(7010):899-909. | Chemotherapy in non-small cell lung cancer: a meta-analysis using updated data on individual patients from 52 randomised clinical trials. |
| 667 | British Medical Journal | 2012;344:e486. | Meta-analysis of individual patient data in randomised trials of self monitoring of blood glucose in people with non-insulin treated type 2 diabetes. |
| 668 | British Medical Journal | 2010;340:b5463. | Patient level pooled analysis of 68 500 patients from seven major vitamin D fracture trials in US and Europe. |
| 669 | British Medical Journal | 2000;321(7269):1113-1116 | Safety and costs of initiating angiotensin converting enzyme inhibitors for heart failure in primary care: analysis of individual patient data from studies of left ventricular dysfunction |
| 670 | Breast Cancer Research & Treatment | 2010;119(3):633-641 | Efficacy of oral tegafur-uracil (UFT) as adjuvant therapy as compared with classical cyclophosphamide, methotrexate, and 5-fluorouracil (CMF) in early breast cancer: a pooled analysis of two randomized controlled trials (NSAS-BC 01 trial and CUBC trial) |
| 671 | Breast Cancer Research & Treatment | 2011;125(3):755-765. | Ixabepilone plus capecitabine in metastatic breast cancer patients with reduced performance status previously treated with anthracyclines and taxanes: a pooled analysis by performance status of efficacy and safety data from 2 phase III studies. |
| 672 | Breast Cancer Research & Treatment | 2011;125(1):145-156 | Impact of treatment characteristics on response of different breast cancer phenotypes: pooled analysis of the German neo-adjuvant chemotherapy trials |
| 673 | British Journal of Cancer | 2004;91(4):683-687 | Metastatic seminoma treated with either single agent carboplatin or cisplatin-based combination chemotherapy: a pooled analysis of two randomised trials |
| 674 | British Journal of General Practice | 2011;61(590):e542-548 | Acute infective conjunctivitis in primary care: who needs antibiotics? An individual patient data meta-analysis |
| 675 | British Journal of Surgery | 2012;99(5):655-665. | Meta-analysis of individual patient data to examine factors affecting growth and rupture of small abdominal aortic aneurysms. |
| 676 | Bulletin of the World Health Organization | 1991;69(5):541-548 | Impact of glycine-containing ORS solutions on stool output and duration of diarrhoea: a meta-analysis of seven clinical trials |
| 677 | Cancer | 2005;104(8):1742-1750 | Survival of metastatic breast carcinoma patients over a 20-year period: a retrospective analysis based on individual patient data from six consecutive studies |
| 678 | Cancer | 2009;115(23):5516-5525 | Venous thromboembolism and nonsmall cell lung cancer: a pooled analysis of National Cancer Institute of Canada Clinical Trials Group trials |
| 679 | Circulation | 2008;118:2038-2046 | Antithrombotic therapy with fondaparinux in relation to interventional management strategy in patients with ST- and non-ST-segment elevation acute coronary syndromes: an individual patient-level combined analysis of the Fifth and Sixth Organization to Assess Strategies in Ischemic Syndromes (OASIS 5 and 6) randomized trials |
| 680 | Circulation | 2012;5(3):309-314 | Renal dysfunction in patients with heart failure with preserved versus reduced ejection fraction: impact of the new Chronic Kidney Disease-Epidemiology Collaboration Group formula |
| 681 | Clinica Chimica Acta | 2011;412(17-18):1521-1526 | Accuracy of a prediction model for heparin-induced thrombocytopenia (HIT): an analysis based on individual patient data |
| 682 | Clinical & Experimental Hypertension (New York) | 1999;21(5-6):517-529 | An overview of 37 randomised trials of blood pressure lowering agents among 270,000 individuals World Health Organization-International Society of Hypertension Blood Pressure Lowering Treatment Trialists' Collaboration |
| 683 | Clinical Infectious Diseases | 2012;54(8):1110-1122 | Impact of treatment strategy on outcomes in patients with candidemia and other forms of invasive candidiasis: a patient-level quantitative review of randomized trials |
| 684 | Clinical Infectious Diseases | 2005;41(8):1105-1113 | Two randomized controlled trials of ceftazidime alone versus ceftazidime in combination with trimethoprim-sulfamethoxazole for the treatment of severe melioidosis |
| 685 | CNS Spectrums | 2009;14(6):326-333 | Escitalopram versus SNRI antidepressants in the acute treatment of major depressive disorder: integrative analysis of four double-blind, randomized clinical trials |
| 686 | Cochrane Database of Systematic Reviews | 2000;(2):CD001418 | Chemotherapy for advanced ovarian cancer |
| 687 | Cochrane Database of Systematic Reviews | 2000(4):CD001768 | Follow-up strategies for women treated for early breast cancer |
| 688 | Cochrane Database of Systematic Reviews | 2009;4:CD001769 | Phenytoin versus valproate monotherapy for partial onset seizures and generalized onset tonic-clonic seizures |
| 689 | Cochrane Database of Systematic Reviews | 2009;4:CD003615 | Oxcarbazepine versus phenytoin monotherapy for epilepsy |
| 690 | Cochrane Database of Systematic Reviews | 2009;4:CD001031 | Lamotrigine versus carbamazepine monotherapy for epilepsy |
| 691 | Cochrane Database of Systematic Reviews | 2010(1):CD008285. | Reducing uncertainties about the effects of chemoradiotherapy for cervical cancer: individual patient data meta-analysis. |
| 692 | Cochrane Database of Systematic Reviews | 2007(2):CD005478 | Naftidrofuryl for acute stroke |
| 693 | Cochrane Database of Systematic Reviews | 2000(3):CD001030 | Carbamazepine versus valproate monotherapy for epilepsy |
| 694 | Cochrane Database of Systematic Reviews | 2011(2):CD008216 | Autologous hematopoietic stem cell transplantation following high-dose chemotherapy for non-rhabdomyosarcoma soft tissue sarcomas |
| 695 | European Journal of Cardiovascular Prevention & Rehabilitation | 2011;18(2):175-85 | Predicting CHD risk in France: a pooled analysis of the D.E.S.I.R., Three City, PRIME, and SU.VI.MAX studies |
| 696 | Current Medical Research & Opinion | 2007;23(6):1245-1252 | Pharmacotherapy of generalized anxiety disorder: results of duloxetine treatment from a pooled analysis of three clinical trials |
| 697 | Current Medical Research & Opinion | 2010;26(7):1621-1628 | Starting insulin therapy with basal insulin analog or premix insulin analog in T2DM: a pooled analysis of treat-to-target trials |
| 698 | AIDS | 1998;12(15):1991-7 | HIV-1 RNA, CD4 cell count and the risk of progression to AIDS and death during treatment with HIV-1 reverse transcriptase inhibitors |
| 699 | Diabetes Care | 2004;27(12):2836-2842 | Blood glucose and risk of cardiovascular disease in the Asia Pacific region |
| 700 | Diabetes Care | 2000 Apr;23 Suppl 2:B65-71. | Efficacy of diuretics and beta-blockers in diabetic hypertensive patients |
| 701 | Diseases of the Esophagus | 2012;25(2):130-136 | Complete response to preoperative chemoradiation and survival in esophageal cancer: A pooled analysis of three single-institution phase II trials |
| 702 | Eurointervention | 2011;7(7):859-871 | Cardiovascular risk profile of patients included in stent trials; a pooled analysis of individual patient data from randomised clinical trials: insights from 33 prospective stent trials in Europe |
| 703 | European Heart Journal | 2007;28(14):1746-1749 | Role of the implantable defibrillator among elderly patients with a history of life-threatening ventricular arrhythmias |
| 704 | European Journal of Cardio-Thoracic Surgery | 2006;30(5):700-705 | Low birth weight or diagnosis, which is a higher risk?--A meta-analysis of observational studies |
| 705 | European Journal of Surgery | 2000;166(9):706-712 | Pooling of prognostic studies in cancer of the pancreatic head and periampullary region: the Triple-P study |
| 706 | European Urology | 2009;56(2):247-256. | An Individual Patient Data Meta-Analysis of the Long-Term Outcome of Randomised Studies Comparing Intravesical Mitomycin C versus Bacillus Calmette-Guerin for Non-Muscle-Invasive Bladder Cancer. |
| 707 | European Urology | 2004;45(3):297-303 | Neoadjuvant cisplatinum based combination chemotherapy in patients with invasive bladder cancer: a combined analysis of two Nordic studies |
| 708 | Evidence based Complementary and Alternative Medicine | 2010;7(2):157-166 | Individual patient data meta-analysis of survival and psychosomatic self-regulation from published prospective controlled cohort studies for long-term therapy of breast cancer patients with a mistletoe preparation (iscador) |
| 709 | Gastroenterology | 1999;117(2):408-413 | Interferon-ribavirin for chronic hepatitis C with and without cirrhosis: analysis of individual patient data of six controlled trials Eurohep Study Group for Viral Hepatitis |
| 710 | Gastrointestinal Endoscopy | 2005;62(3):383-389 | Cyst fluid analysis in the differential diagnosis of pancreatic cystic lesions: a pooled analysis |
| 711 | Health Technology Assessment | 2010;14(Suppl 2):11-17 | Capecitabine for the treatment of advanced gastric cancer |
| 712 | Hematological Oncology | 2011;29(4):177-184 | Pegfilgrastim primary prophylaxis in patients with non-Hodgkin lymphoma: results from an integrated analysis |
| 713 | Hepatology | 1994;19(4):820-828 | Interferon-alpha for chronic hepatitis C: an analysis of pretreatment clinical predictors of response |
| 714 | International Journal of Cancer | 1995;62(4):367-376 | Risk of cutaneous melanoma associated with pigmentation characteristics and freckling: systematic overview of 10 case-control studies The International Melanoma Analysis Group (IMAGE) |
| 715 | International Journal of Cardiology | 2008;126(1):101-107 | Potential demographic and baselines variables for risk stratification of high-risk post-myocardial infarction patients in the era of implantable cardioverter-defibrillator--a prognostic indicator |
| 716 | International Journal of Clinical Pharmacology & Therapeutics | 2011;49(11):637-647 | Efficacy and tolerability of flupirtine in subacute/ chronic musculoskeletal pain - results of a patient level, pooled re-analysis of randomized, double-blind, controlled trials |
| 717 | International Journal of Epidemiology | 2004;33(4):751-758 | Body mass index and cardiovascular disease in the Asia-Pacific Region: an overview of 33 cohorts involving 310 000 participants |
| 718 | International Journal of Epidemiology | 2005;34(5):1036-1045 | Smoking, quitting, and the risk of cardiovascular disease among women and men in the Asia-Pacific region |
| 719 | International Journal of Epidemiology | 2003;32(4):563-572 | Cholesterol, coronary heart disease, and stroke in the Asia Pacific region |
| 720 | International Journal of Radiation Oncology*Biology*Physics | 2003;55(3):568-575 | Compliance to the prescribed dose and overall treatment time in five randomized clinical trials of altered fractionation in radiotherapy for head-and-neck carcinomas |
| 721 | The Journal of the American Medical Association | 2012;307(12):1302-1309 | Association of LDL cholesterol, non-HDL cholesterol, and apolipoprotein B levels with risk of cardiovascular events among patients treated with statins: a meta-analysis |
| 722 | The Journal of the American Medical Association | 2002;288(16):2023-2031. | MTHFR 677C-->T polymorphism and risk of coronary heart disease: a meta-analysis. |
| 723 | Journal of Clinical Oncology | 2007;25(33):5218-5224 | Progression-free survival is a surrogate for survival in advanced colorectal cancer |
| 724 | Journal of Clinical Oncology | 2008;26(21):3573-3581 | Pooled analysis of the effect of age on adjuvant cisplatin-based chemotherapy for completely resected non-small-cell lung cancer |
| 725 | Journal of Clinical Oncology | 2012;30(13):1541-1549. | Serum mesothelin for diagnosing malignant pleural mesothelioma: an individual patient data meta-analysis. |
| 726 | Journal of Clinical Oncology | 2009;27(17):2831-2837 | Newer antidepressants and gabapentin for hot flashes: an individual patient pooled analysis |
| 727 | Journal of Clinical Oncology | 2008;26(21):3552-3559 | Lung adjuvant cisplatin evaluation: a pooled analysis by the LACE Collaborative Group |
| 728 | Journal of Clinical Oncology | 2012;30(14):1692-1698 | Carboplatin- or cisplatin-based chemotherapy in first-line treatment of small-cell lung cancer: the COCIS meta-analysis of individual patient data |
| 729 | Journal of Clinical Oncology | 2005;23(34):8664-8670 | Disease-free survival versus overall survival as a primary end point for adjuvant colon cancer studies: individual patient data from 20,898 patients on 18 randomized trials |
| 730 | Journal of Hypertension | 2007;25(6):1205-1213 | Systolic blood pressure, diabetes and the risk of cardiovascular diseases in the Asia-Pacific region |
| 731 | Journal of Hypertension | 2003;21(4):707-716 | Blood pressure and cardiovascular disease in the Asia Pacific region |
| 732 | Journal of Neurotrauma | 2007;24(2): 281-286. | Prognostic value of cause of injury in traumatic brain injury: results from the IMPACT study |
| 733 | Journal of Pediatrics | 2011;159(4): 644-651. | Procalcitonin is a Predictor for High-Grade Vesicoureteral Reflux in Children: Meta-Analysis of Individual Patient Data (The Journal of Pediarics (2011) 159 (644-651)) |
| 734 | Journal of Public Health | 2011;19(6):563-568 | Effectiveness of guidelines in treatment of allergic rhinitis: An analysis of individual patient data |
| 735 | Journal of the American College of Cardiology | 2000;35(7):1801-1807 | Clinical effects of early angiotensin-converting enzyme inhibitor treatment for acute myocardial infarction are similar in the presence and absence of aspirin: systematic overview of individual data from 96,712 randomized patients Angiotensin-converting Enzyme Inhibitor Myocardial Infarction Collaborative Group |
| 736 | Journal of the European Academy of Dermatology & Venereology | 2012;26(2):231-235. | Efficacy of tumour necrosis factor-alpha antagonists in aphthous ulceration: review of published individual patient data |
| 737 | Journal of Thrombosis & Haemostasis | 2008;6(10):1713-1719 | Differences in clinical presentation of deep vein thrombosis in men and women |
| 738 | The Lancet | 2004;363(9402):9-17 | Artesunate combinations for treatment of malaria: meta-analysis |
| 739 | The Lancet | 2002;359(9302):189-198. | Platelet glycoprotein IIb/IIIa inhibitors in acute coronary syndromes: a meta-analysis of all major randomised clinical trials. |
| 740 | The Lancet | 2010;375(9731):2082-2091 | Elective high-frequency oscillatory versus conventional ventilation in preterm infants: a systematic review and meta-analysis of individual patients' data |
| 741 | The Lancet | 2002;359(9303):294-302 | Direct thrombin inhibitors in acute coronary syndromes: principal results of a meta-analysis based on individual patients' data |
| 742 | The Lancet | 2012;379(9814):432-444. | Comparisons between different polychemotherapy regimens for early breast cancer: meta-analyses of long-term outcome among 100,000 women in 123 randomised trials. |
| 743 | The Lancet | 2004;363(9411):768-774 | Association of outcome with early stroke treatment: pooled analysis of ATLANTIS, ECASS, and NINDS rt-PA stroke trials |
| 744 | The Lancet | 2009;373(9670):1190-1197. | Coronary artery bypass surgery compared with percutaneous coronary interventions for multivessel disease: a collaborative analysis of individual patient data from ten randomised trials. |
| 745 | The Lancet | 2012;379(9831):2053-2062. | Carotid intima-media thickness progression to predict cardiovascular events in the general population (the PROG-IMT collaborative project): a meta-analysis of individual participant data |
| 746 | The Lancet | 2012;379(9826):1602-1612. | Short-term effects of daily aspirin on cancer incidence, mortality, and non-vascular death: analysis of the time course of risks and benefits in 51 randomised controlled trials |
| 747 | The Lancet | 2010;376(9754):1741-1750. | Long-term effect of aspirin on colorectal cancer incidence and mortality: 20-year follow-up of five randomised trials. |
| 748 | The Lancet Oncology | 2010;11(12):1160-1171 | Performance of high-risk human papillomavirus DNA testing as a primary screen for cervical cancer: a pooled analysis of individual patient data from 17 population-based studies from China |
| 749 | Leukemia & Lymphoma | 2002;43(12):2301-2307 | Thalidomide in myelofibrosis with myeloid metaplasia: a pooled-analysis of individual patient data from five studies |
| 750 | Movement Disorders | 2008;23(5):690-699 | Placebo response in Parkinson's disease: comparisons among 11 trials covering medical and surgical interventions |
| 751 | Movement Disorders | 2011;26(2):209-215 | Dose response with onabotulinumtoxinA for post-stroke spasticity: a pooled data analysis |
| 752 | Neurosurgery | 2012;70(4):811-818. | Prognostic value of major extracranial injury in traumatic brain injury: An individual patient data meta-analysis in 39 274 patients |
| 753 | BMC cancer | 2008;8:82. | Meta-analysis of randomized trials: evaluation of benefits from gemcitabine-based combination chemotherapy applied in advanced pancreatic cancer. |
| 754 | Pain | 1996;66(2-3):229-237 | Deriving dichotomous outcome measures from continuous data in randomised controlled trials of analgesics |
| 755 | Pediatrics | 2011;128(3):e550-564 | Estimating the probability of abusive head trauma: a pooled analysis |
| 756 | Pharmaceutical Statistics | 2004;3(3):205-212 | The relationship between osteoporotic fracture risk and a surrogate: Apparent discrepancies between analyses based on individual patient data and summary statisrics |
| 757 | PLoS Medicine | 2008;5(9):e193 | Birth size and breast cancer risk: re-analysis of individual participant data from 32 studies |
| 758 | PLoS One | 2012;7(7) | Job strain and alcohol intake: A collaborative meta-analysis of individual-participant data from 140 000 men and women |
| 759 | PLoS One | 2012;7(7) | Job strain and tobacco smoking: An individual-participant data meta-analysis of 166 130 adults in 15 european studies |
| 760 | PLoS One | 2012;7(3):e30325. | Performance of biomarkers FibroTest, ActiTest, SteatoTest, and NashTest in patients with severe obesity: meta analysis of individual patient data |
| 761 | PLoS One | 2009;4(7):e6358 | Safety and efficacy of dihydroartemisinin-piperaquine in falciparum malaria: a prospective multi-centre individual patient data analysis |
| 762 | Preventive Medicine | 2011;52(1):60-65 | Population attributable numbers and fractions of deaths due to smoking: a pooled analysis of 180,000 Japanese |
| 763 | Radiotherapy and Oncology | 2009;92(1):4-14. | Meta-analysis of chemotherapy in head and neck cancer (MACH-NC): an update on 93 randomised trials and 17,346 patients. |
| 764 | Journal of Epidemiology | 2008;18(6):251-64 | Population attributable fraction of mortality associated with tobacco smoking in Japan: a pooled analysis of three large-scale cohort studies |
| 765 | Stroke | 2012;43(6):1496-1504. | Individual patient data subgroup meta-analysis of surgery for spontaneous supratentorial intracerebral hemorrhage |
| 766 | Stroke | 2011;42(10):2825-2831 | Decompressive surgery in cerebrovenous thrombosis: A multicenter registry and a systematic review of individual patient data |
| 767 | Stroke | 2004;35(3):710-714 | Risk of intracerebral hemorrhage in patients with arterial versus cardiac origin of cerebral ischemia on aspirin or placebo: analysis of individual patient data from 9 trials |
| 768 | Stroke | 2000;31(6):1240-1249 | Indications for early aspirin use in acute ischemic stroke : A combined analysis of 40 000 randomized patients from the chinese acute stroke trial and the international stroke trial On behalf of the CAST and IST collaborative groups |
| 769 | Stroke | 2008;39(6):1694-1702 | Cigarette smoking, systolic blood pressure, and cardiovascular diseases in the Asia-Pacific region |
| 770 | Vaccine | 2011;29(35):5869-5885 | A systematic review of experimental infections with enterotoxigenic Escherichia coli (ETEC) |
| 771 | Value in Health | 2012;15(3):524-533. | Efficacy of once-daily indacaterol relative to alternative bronchodilators in COPD: a patient-level mixed treatment comparison |
| 772 | Biomedical and Environmental Sciences | 2001;14(4):341-9 | The effect of antihypertensive treatment on cardiovascular events--a meta-analysis of four clinical trials in China |
| 773 | British Journal of Cancer | 2000;83(5):692-698 | A pooled analysis of magnetic fields and childhood leukemia |
| 774 | Journal of Clinical Oncology | 1991;9(9):1668-74 | Cyclophosphamide plus cisplatin versus cyclophosphamide, doxorubicin, and cisplatin chemotherapy of ovarian carcinoma: a meta-analysis. The Ovarian Cancer Meta-Analysis Project. |
| 775 | The Lancet | 2001;358:781-86 | Survival and recurrence after concomitant chemotherapy and radiotherapy for cancer of the uterine cervix: a systematic review and meta-analysis |
| 776 | European Heart Journal | 2000;21:2071-2078 | Meta-analysis of the implantable cardioverter defibrillator secondary prevention trials |
| 777 | British Journal of Cancer | 2010;103(7):1128-35. | Pooled analysis of recent studies on magnetic fields and childhood leukaemia |
| 778 | Journal of Clinical Oncology | 2005;23:4999-5006 | meta-analysis of postoperative adjuvant chemotherapy with tegafur-uracil in non-small-cell lung cancer |
| 779 | Journal of Urology | 2004;171:561-569 | Neoadjuvant chemotherapy for transitional cell carcinoma of the bladder: a systematic review and meta-analysis |
| 780 | British Medical Journal | 1994;308(6921):81-106. | Collaborative overview of randomised trials of antiplatelet therapy prevention of death, myocardial infarction, and stroke by prolonged antiplatelet therapy in various categories of patients |
| 781 | Journal of Neurotrauma | 2007;24(2): 329-337. | Multivariable prognostic analysis in traumatic brain injury: results from the IPMACT study |
| 782 | Journal of Neurotrauma | 2007;24(2): 294-302. | Prognostic value of admission blood pressure in traumatic brain injury: resluts from the IMPACT study. |
| 783 | Journal of Neurotrauma | 2007;24(2): 315-328. | Prongnstic value of admission laboratory parameters in traumatic brain injury: results from the IMPACT study. |
| 784 | Journal of Neurotrauma | 2007;24(2): 303-314. | Prognostic value of computerized tomography scan characteristics in traumatic brain injury: results from the IMAPCT study. |
| 785 | Journal of Neurotrauma | 2007;24(2): 359-269. | Prognostic value of demographic characteristics in traumatic brain injury: results from the IMPACT study. |
| 786 | Journal of Neurotrauma | 2007;24(2): 287-293. | Prognostic value of secondary insults in traumatic brain injury: results from the IMPACT study. |
| 787 | Journal of Neurotrauma | 2007;24(2): 270-280. | Prognostic value of the Glasgow Coma Scale and pupil reactivity in traumatic brain injury assessed pre-hospital and on enrollment: an IMPACT analysis. |
| 788 | Journal of Neurotrauma | 2007;24(2): 251-258. | Statistical approaches to the univariate prognostic analysis of the IMPACT database on traumatic brain injury. |
| 789 | Neurocritical Care | 2007;6(3): 181-185. | Safety outcomes of Alteplase among acute ischemic stroke patients with special characteristics. |
| 790 | Blood | 2006;108(9): 2937-2941. | Gender impalance and risk factor interactions in heparin-induced thrombocytopenia. |
| 791 | Diabetes, Obesity and Metabolism | 2012;14(1): 77-82. | Achieving a clinically relevant composite outcome of an HbA1c of <7% without weight gain or hypoglycaemia in type 2 diabetes: a meta-analysis of the liraglutide clinical trial programme. |
| 792 | European Journal of Cancer | 2012;48(10): 1466-1475. | Addition of cetuximab to chemotherapy as first-line treatment for KRAS wild-type metastatic colorectal cancer: Pooled analysis of the CRYSTAL and OPUS randomised clinical trials |
| 793 | Annals of Oncology | 2012;23(2): 374-382. | Alcohol consumption and pancreatic cancer: a pooled analysis in the International Pancreatic Cancer Case-Control Consortium. |
| 794 | European Heart Journal | 2012;33(4): 505-514. | Angiotensin-converting enzyme inhibitors or angiotensin receptor blockers are beneficial in normotensive atherosclerotic patients: A collaborative meta-analysis of randomized trials |
| 795 | Pacing & Clinical Electrophysiology | 2012;35(2): 131-134. | Are elderly patients at increased risk of complications following pacemaker implantation? A meta-analysis of randomized trials |
| 796 | Headache | 2012;52(1): 48-56. | Aspirin is first-line treatment for migraine and episodic tension-type headache regardless of headache intensity. |
| 797 | The Lancet Oncology | 2012;13(4): 358-394. | Association between endometriosis and risk of histological subtypes of ovarian cancer: a pooled analysis of case-control studies |
| 798 | British Medical Journal | 2012;345: e4933. | Association between psychological distress and mortality: individual participant pooled analysis of 10 prospective cohort studies |
| 799 | Journal of Clinical Oncology | 2012;30(19): 2334-2339. | Benefits and adverse events in younger versus older patients receiving adjuvant chemotherapy for colon cancer: Findings from the adjuvant colon cancer endpoints data set |
| 800 | Archives of General Psychiatry | 2012;69(6): 572-579. | Benefits from antidepressants: synthesis of 6-week patient-level outcomes from double-blind placebo-controlled randomized trials of fluoxetine and venlafaxine |
| 801 | European Heart Journal | 2012;33(10): 1214-1222. | Biodegradable polymer drug-eluting stents reduce the risk of stent thrombosis at 4 years in patients undergoing percutaneous coronary intervention: A pooled analysis of individual patient data from the ISAR-TEST 3, ISAR-TEST 4, and LEADERS randomized trials |
| 802 | PLoS One | 2012;7(2): e28510. | Children who acquire HIV infection perinatally are at higher risk of early death than those acquiring infection through breastmilk: a meta-analysis |
| 803 | Medicine (United States) | 2012;91(4): 179-194. | Choroidal metastasis as a presenting manifestation of lung cancer: A report of 3 cases and systematic review of the literature |
| 804 | Malaria Journal | 2012;11(260) | Clinical tolerability of artesunate-amodiaquine versus comparator treatments for uncomplicated falciparum malaria: An individual-patient analysis of eight randomized controlled trials in sub-Saharan Africa |
| 805 | BMC Public Health | 2012;12: 62. | Comparison of alternative versions of the job demand-control scales in 17 European cohort studies: the IPD-Work consortium |
| 806 | Thorax | 2012;67(10): 920-924. | The effect of continuous positive airway pressure usage on sleepiness in obstructive sleep apnoea: real effects or expectation of benefit? |
| 807 | Heart | 2012;98(3): 207-213. | Effects of age on long-term outcomes after a routine invasive or selective invasive strategy in patients presenting with non-ST segment elevation acute coronary syndromes: a collaborative analysis of individual data from the FRISC II - ICTUS - RITA-3 (FIR) trials |
| 808 | Hypertension | 2012;59(6): 1118-1123. | Effects of prehypertension and hypertension subtype on cardiovascular disease in the Asia-pacific region |
| 809 | Depression and Anxiety | 2012;29(7): 574-586. | Extended release quetiapine fumarate in major depressive disorder: Analysis in patients with anxious depression |
| 810 | European Journal of Heart Failure | 2012;14(5): 473-479. | Gender and survival in patients with heart failure: Interactions with diabetes and aetiology. Results from the MAGGIC individual patient meta-analysis |
| 811 | Diabetes Care | 2012;35(3): 556-564. | Global prevalence and major risk factors of diabetic retinopathy |
| 812 | European Urology | 2005;48(2): 202-206. | Neoadjuvant chemotherapy in invasive bladder cancer: update of a systematic review and meta-analysis of individual patient data advanced bladder cancer (ABC) meta-analysis collaboration. |
| 813 | Journal of Clinical Oncology | 2012;30(22): 2788-2797. | Hyperfractionated or accelerated radiotherapy in lung cancer: An individual patient data meta-analysis |
| 814 | Value in Health | 2012;15(1): 175-182. | Impact of secondary cardiovascular events on health status |
| 815 | JACC cardiovascluar interventions | 2012;5(3): 301-310. | Impact of sex on clinical and angiographic outcomes among patients undergoing revascularization with drug-eluting stents |
| 816 | European Journal of Preventive Cardiology | 2012;19(3): 428-435. | Individual patient meta-analysis of exercise training effects on systemic brain natriuretic peptide expression in heart failure |
| 817 | PLoS One | 2012;7(1): e29849. | Lack of effect of lowering LDL cholesterol on cancer: meta-analysis of individual data from 175,000 people in 27 randomised trials of statin therapy. |
| 818 | Atherosclerosis | 2012;223(2): 251-261. | Lipid-altering efficacy of ezetimibe plus statin and statin monotherapy and identification of factors associated with treatment response: a pooled analysis of over 21,000 subjects from 27 clinical trials |
| 819 | Malaria Journal | 2012;11(147) | Multiple treatment comparisons in a series of anti-malarial trials with an ordinal primary outcome and repeated treatment evaluations |
| 820 | Annals of Internal medicine | 2012;9(4): e1001200. | Ovarian cancer and body size: individual participant meta-analysis including 25,157 women with ovarian cancer from 47 epidemiological studies. |
| 821 | New England Journal of Medicine | 2012;367(1): 40-49. | A pooled analysis of vitamin D dose requirements for fracture prevention |
| 822 | Journal of Thrombosis & Haemostasis | 2012;10(6): 1019-1025. | Predicting disease recurrence in patients with previous unprovoked venous thromboembolism: A proposed prediction score (DASH) |
| 823 | British Medical Journal | 2012;244(7862) | Prediction model to estimate presence of coronary artery disease: Retrospective pooled analysis of existing cohorts |
| 824 | cancer | 2012;118(9): 2525-2531. | Preliminary indication of survival benefit from ERCC1 and RRM1-tailored chemotherapy in patients with advanced nonsmall cell lung cancer: Evidence from an individual patient analysis |
| 825 | Congestive Heart Failure | 2012;18(4): 198-204. | Prognostic Implications of Left Ventricular Dilation in Patients With Nonischemic Heart Failure: Interactions With Restrictive Filling Pattern and Mitral Regurgitation |
| 826 | Lung Cancer | 2012;77(1): 116-120. | Prognostic score for second-line chemotherapy of advanced non-small-cell lung cancer: External validation in a phase III trial comparing vinflunine with docetaxel |
| 827 | European Journal of Cancer | 2012;48(3): 360-367. | The prophylactic use of granulocyte-colony stimulating factor during remission induction is associated with increased leukaemia-free survival of adults with acute lymphoblastic leukaemia: A joint analysis of five randomised trials on behalf of the EWALL |
| 828 | Journal of Clinical Endocrinology & Metabolism | 2012;97(8): 2670-2681. | Vitamin D with calcium reduces mortality: Patient level pooled analysis of 70,528 patients from eight major vitamin D trials |
| 829 | European Journal of Cancer | 2012;48(12): 1781-1790. | What is the clinical benefit of preoperative chemoradiotherapy with 5FU/leucovorin for T3-4 rectal cancer in a pooled analysis of EORTC 22921 and FFCD 9203 trials: Surrogacy in question? |
